# Current and Future Distributions of *Allium karataviense* and *A. turkestanicum* in Central Asia Using MaxEnt Modeling

**DOI:** 10.1002/ece3.74315

**Published:** 2026-09-09

**Authors:** Alan Ibraimov, Aruzhan Alikhanova, Saule Abugalieva, Shyryn Almerekova

**Affiliations:** ^1^ Institute of Plant Biology and Biotechnology Almaty Kazakhstan

**Keywords:** *Allium karataviense*, *Allium turkestanicum*, Central Asia, climate change, conservation strategies, Kazakhstan, MaxEnt modeling

## Abstract

The genus *Allium* L. represents one of the most diverse groups of monocotyledonous plants in Central Asia, a region recognized as a major center of its diversity. However, ongoing climate change poses a serious threat to the distribution and persistence of many wild species, including *Allium*. In this study, we applied Maximum Entropy (MaxEnt) modeling to assess the current and future potential distributions of *Allium karataviense* Regel and *A. turkestanicum* Regel under three climate change scenarios (ssp126, ssp245, ssp585) and three time periods (2030s, 2070s, and 2090s). The models demonstrated high predictive performance, with AUC values of 0.957 for *A. karataviense* and 0.935 for *A. turkestanicum* . Results of environmental variable contributions showed that *A. karataviense* adapted to mountainous environments, while *A. turkestanicum* adapted to arid lowland habitats. Future distribution areas revealed contrasting responses to climate change. *A. karataviense* is expected to maintain or moderately expand its suitable habitats, whereas *A. turkestanicum* shows a consistent decline in total suitable area across all scenarios. Centroid migration analysis confirmed the stability of *A. karataviense* and the sensitivity of *A. turkestanicum* to climatic changes. The overlap between highly suitable habitats and existing protected areas remains limited. Based on the modeling results, the Western Tien Shan, including the Karatau and Chu‐Ili Mountains, is identified as a potential Important Plant Area (IPA), where suitable habitats of both species overlap. Overall, this study highlights the diverse responses of plant species to climate change and emphasizes the need for targeted, climate‐informed conservation strategies to protect Central Asian wild plant diversity.

## Graphical abstract

The study used Maximum Entropy (MaxEnt) modeling to assess the current and future potential distributions of Allium karataviense Regel and *A. turkestanicum* Regel. The modeling was conducted under three climate change scenarios (SSP1‐2.6, SSP2‐4.5, and SSP5‐8.5) across three future periods, 2030s, 2070s, and 2090s. Based on the results, the Western Tien Shan, including the Karatau and Chu‐Ili Mountains, was identified as a potential Important Plant Area (IPA), where suitable habitats for both species overlap.

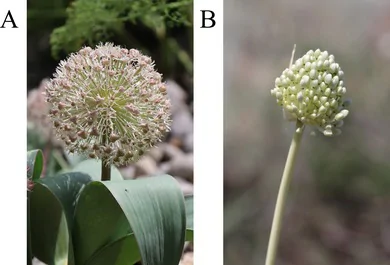

## 1 Introduction

The genus *Allium* L. (Amaryllidaceae J.St.‐Hil.) stands out as one of the most diverse and evolutionarily remarkable groups among monocotyledonous plants, comprising over 1000 species primarily distributed across the Northern Hemisphere (Stearn 1978; Fritsch and Friesen 2002; Chase et al. 2006; Govaerts et al. 2026). This genus includes not only globally important crops such as onion, garlic, and leek, but also a wide array of wild species that harbor significant genetic diversity, ecological importance, and ornamental value (Keusgen et al. 2006; Abugalieva et al. 2017; Wu et al. 2025). Beyond their well‐known culinary value, *Allium* species are rich in biologically active compounds, organosulfur compounds, and flavonoids, which are associated with notable medicinal properties, including antimicrobial, antioxidant, and cardioprotective effects (Bastaki et al. 2021). In natural ecosystems, wild *Allium* species play a crucial ecological role, serving as early‐season nectar sources for pollinators and contributing to the stability and functioning of plant communities, especially in arid and mountainous regions (Kamenetsky and Gutterman 2000; Zito et al. 2019; Vesselova et al. 2025).

The area from the Mediterranean basin to Central Asia is widely recognized as one of the two main diversity centers of the genus *Allium* (Fritsch and Friesen 2002; Xie et al. 2020), representing a hotspot of evolutionary processes, species distribution, and endemism (Fritsch 2016). Central Asia encompasses an exceptional range of landscapes: from arid deserts and semi‐deserts to vast steppes and complex mountain systems such as the Tien Shan and Pamir‐Alai, forming a mosaic of ecological niches that promote isolation, adaptation, and speciation (Baitulin 2006; Tayjanov et al. 2017). The pronounced environmental heterogeneity, coupled with a dynamic geological history and past climatic fluctuations, has driven intensive in situ diversification and the formation of numerous wild endemic taxa (Hauenschild et al. 2017; Ma et al. 2024). As a result, Central Asia is recognized not only as a major center of biodiversity for the genus *Allium* (Fritsch and Friesen 2002), but also for several other taxonomically significant genera, including *Tulipa* L. (Tojibaev and Beshko 2015), *Iris* L. (Khassanov and Rakhimova 2012), *Atraphaxis* L. (Zhang et al. 2014), *Gagea* Salisb. (Peterson et al. 2016), *Hedysarum* L. (Nafisi et al. 2019), and *Eremurus* M.Bieb. (Makhmudjanov et al. 2024), many of which contain a high proportion of threatened and endemic species (Takhtajan 1978; Zhang et al. 2020; Zhang, Zhang, et al. 2013; Ma et al. 2024). This highlights the exceptional floristic value of Central Asia and its importance as a global reservoir of unique genetic resources.

Within this broader Central Asian context, Kazakhstan represents a core component of this diversification center (Yermagambetova et al. 2026), being the largest Central Asian country with a total area of 2.7 million km^2^. The country is landlocked and has unique flora comprising about 6000 vascular plant species, with approximately 10% endemic to this region (Pavlov 1961). The distribution of endemic plant species varies markedly across its floristic regions, reflecting the spatial heterogeneity of biodiversity patterns. The diversity hotspots are primarily located in the mountainous regions of southern, southeastern, and eastern Kazakhstan, including the northern, northwestern, and western parts of the Tien Shan and the Altay Mountains (Pavlov 1961; Ryabushkina et al. 2016; Shaimoldina et al. 2026). Each of these systems exhibits a unique botanical composition, including representatives of *Allium*.

Among the Central Asian representatives of the genus *Allium*, *A. karataviense* Regel and *A. turkestanicum* Regel are of particular scientific and conservation interest, differing in morphology, ecology, and geographic distribution, yet united by their adaptation to arid continental environments. These species belong to different evolutionary lineages within the genus: *A. karataviense* to the second evolutionary line in the subgenus *Melanocrommyum* (Webb et Berthel.) Rouy, while *A. turkestanicum* belongs to the subgenus *Allium* L., within the third evolutionary line (Friesen et al. 2006; Friesen 2022). Importantly, both species are also type species of their respective sections: *A. karataviense* is the type species of section *Miniprason* R.M.Fritsch, whereas *A. turkestanicum* serves as the type species of section *Mediasia* F.O.Khass., Yengalycheva & N.Friesen (Friesen et al. 2006; Friesen 2022; Almerekova et al. 2026).

The species *A. karataviense* typically grows on unstable screes and gravelly substrates in the lower and middle mountain belts. Its distribution is largely confined to southern Kazakhstan, including the Chu‐Ili Mountains, Karatau Mountains, and Western Tien Shan (Pavlov 1947), while its broader range extends into Kyrgyzstan, Tajikistan, Uzbekistan, and Afghanistan (Vesselova et al. 2025; POWO 2026). Morphologically, it is distinguished by broad, glaucous leaves and compact, dense inflorescences, which contribute to its high ornamental value (Fritsch et al. 2007). At the same time, its bulbs contain high concentrations of saponins and are considered toxic, highlighting its biochemical specificity and potential pharmacological relevance, with wound‐healing, antibacterial, and antispasmodic properties (Pavlov 1947; Vollerner et al. 1980; Kuroda et al. 2015). In contrast, *A. turkestanicum* occupies a wider range of habitats, adapting to more arid and open landscapes, including clayey, sandy, and gravelly desert steppes. It is distributed across multiple floristic regions of Kazakhstan, such as Kyzyl‐Orda, Betpakdala, Balkhash‐Alakol, Turkestan, Trans‐Ili Kungei Alatau, Chu‐Ili Mountains, Karatau Mountains, and Western Tien Shan (Pavlov 1947), with a general distribution covering Kyrgyzstan, Tajikistan, Turkmenistan, and Uzbekistan (Vesselova et al. 2025; POWO 2026). *A. turkestanicum* 's small bulbs are edible, which determines its potential economic importance (Baitulin et al. 2012). *A. karataviense* and *A. turkestanicum* are perennial bulbous geophytes with a typical spring ephemeroid life cycle. Both species occur in open habitats exposed to full sunlight. Vegetative growth begins shortly after snowmelt, when soil moisture is highest, and leaves remain photosynthetically active during early spring. Flowering generally occurs from April to May, followed by fruit maturation in late spring or early summer. After seed dispersal, the aerial parts senesce, and the plants enter summer dormancy, surviving unfavorable hot and dry conditions as underground bulbs (Kamenetsky 1994, 1996; Khodorova and Boitel‐Conti 2013).

However, despite its global biogeographical significance, Central Asia is also one of the regions vulnerable to ongoing climate change (Lioubimtseva and Henebry 2009; Fallah et al. 2024). The consequences of global warming, including altered precipitation regimes, rising temperatures, soil aridity, and stratospheric ozone depletion, are rapidly transforming ecosystems worldwide, reshaping species distributions and disrupting natural populations and community structures (Kamp et al. 2016). These processes are particularly pronounced in Central Asia, where observed temperature increases exceed the global average, intensifying aridity and accelerating ecosystem degradation (Chen et al. 2009; Reyer et al. 2017). Thus, understanding the relationships between climatic variables and species distributions is of critical importance for Central Asia. Notably, the genus *Allium*, alongside *Astragalus* L. and *Tulipa*, contains the highest number of threatened species among vascular plants in the flora of Kazakhstan (Kamp et al. 2016; Shaimoldina et al. 2026). They are especially vulnerable to habitat degradation and climate‐driven range shifts caused by environmental change and anthropogenic pressure (Zhang, Li, et al. 2013; Li et al. 2015).

To assess and predict these potential impacts, bioclimate models are frequently employed. Also known as species distribution models (SDM) or ecological niche models (ENM), these approaches use climatic variables and other predictors, such as land‐cover data, to estimate habitat suitability across landscapes (Xu et al. 2019). The outputs of such models are typically habitat suitability maps, which can then be used to infer future population trends. For example, by comparing current and projected suitability maps for three generations into the future, researchers can estimate expected population reductions and assess extinction risk under the International Union for Conservation of Nature (IUCN) criteria (IUCN Standards and Petitions Committee 2024). In the context of Central Asia, such modeling is particularly valuable for guiding conservation strategies for *Allium* species and other taxa highly sensitive to climatic shifts.

Among the various modeling approaches, Maximum Entropy Modeling (MaxEnt) has become widely used for its robustness and effectiveness with presence‐only data, which is particularly important for rare or narrowly distributed species (Phillips, Anderson, and Schapire 2006; Tsoar et al. 2007; Lv and Zhou 2018; Xu et al. 2019). MaxEnt estimates the probability distribution of a species' occurrence by maximizing entropy while constraining predictions to match environmental conditions at known occurrence sites, producing continuous habitat suitability maps ranging from unsuitable to highly suitable. The method handles multiple environmental variables simultaneously, captures non‐linear relationships, is robust with small sample sizes, and provides interpretable outputs such as response curves and variable importance scores. Furthermore, MaxEnt allows projections under future climate scenarios, enabling researchers to identify potential conservation areas and assess the risk of range contraction or local extinction (Phillips, Anderson, and Schapire 2006).

Present and future distribution patterns of threatened Central Asian *Allium* species are still poorly characterized, and the impact of environmental factors on their habitat suitability under climate change has not been fully assessed (Ergashov et al. 2025). MaxEnt‐based modeling offers a powerful tool for solving these problems and guiding conservation strategies. Several studies have already applied MaxEnt to *Allium* species, focusing on both widespread and rare taxa. For instance, a study by Ergashov et al. (2025) evaluates the impact of climate change on the distribution of ten threatened *Allium* species (*A. pskemense, A. viridiflorum, A*. *majus* , *A. praemixtum, A*. *giganteum* , *A. isakulii, A. backhousianum, A. eremoprasum, A. aflatunense*, and *A. alaicum*) in Central Asia (Ergashov et al. 2025). Similar approaches have been applied in the Central Zagros of Iran, where Nasab and Zeraatkar (2025) used MaxEnt to assess the potential impacts of global warming on *A. stipitatum* (Nasab and Zeraatkar 2025). Also, Huang et al. (2025) used MaxEnt modeling to evaluate changes in the potential distribution and suitable cultivation areas of *A. victorialis* , a wild vegetable with significant edible and medicinal value under future climate scenarios (Huang et al. 2025). In Kazakhstan, such studies remain limited. To date, there are no comprehensive assessments on the distribution patterns of Kazakhstani *Allium* species using MaxEnt or any other ENM, indicating an important research gap.

To move toward more large‐scale, applied conservation actions, the concept of Important Plant Areas (IPAs) offers a valuable framework for identifying and prioritizing key sites for plant‐diversity conservation. The conception of IPAs was developed by Plantlife International in 2004 (Plantlife International 2004), modified by Darbyshire et al. in 2017 (Darbyshire et al. 2017), and further adapted by Dubynin and Dimeyeva in 2025, who established more flexible criteria for identifying IPAs in Kazakhstan (Dubynin and Dimeyeva 2025). According to Dubynin and Dimeyeva (2025), sites are selected based on three criteria: threatened species (A), botanical diversity (B), and endangered habitats (C). Notably, this framework considers not only biological but also social and economic importance, alongside vulnerability to climatic and anthropogenic pressures (Dubynin and Dimeyeva 2025). IPAs are among the most effective programs in existing global plant conservation strategies, including the Convention on Biological Diversity (CBD) (The Convention on Biological Diversity (CBD) 2026) and the IUCN Species Survival Commission (SSC) (IUCN Species Survival Commission (SSC) 2026). In Kazakhstan, more IPAs have been appearing recently. For instance, IPA “Kurty Valley” includes the habitats of the endemics *T. regelii* Krasn. and *I. kuschakewiczii* Regel (Dubynin 2023), and IPA “Petrophytic Steppes of Ulytau”, which covers the habitat of rare *Tanacetum ulutavicum* Tzvelev (Izbassarova et al. 2026).

In this study, we assess the potential impacts of climate change on the distribution of *A. karataviense* and *A. turkestanicum* in Central Asia using MaxEnt modeling. By combining species occurrence data with climatic and environmental variables, we aim to identify current suitable habitats, predict future range shifts under different climate scenarios, and highlight regions that may serve as potential IPAs for these species.

## 2 Materials and Methods

### 2.1 Collection and Analysis of Species Occurrence Data

**FIGURE 1:**
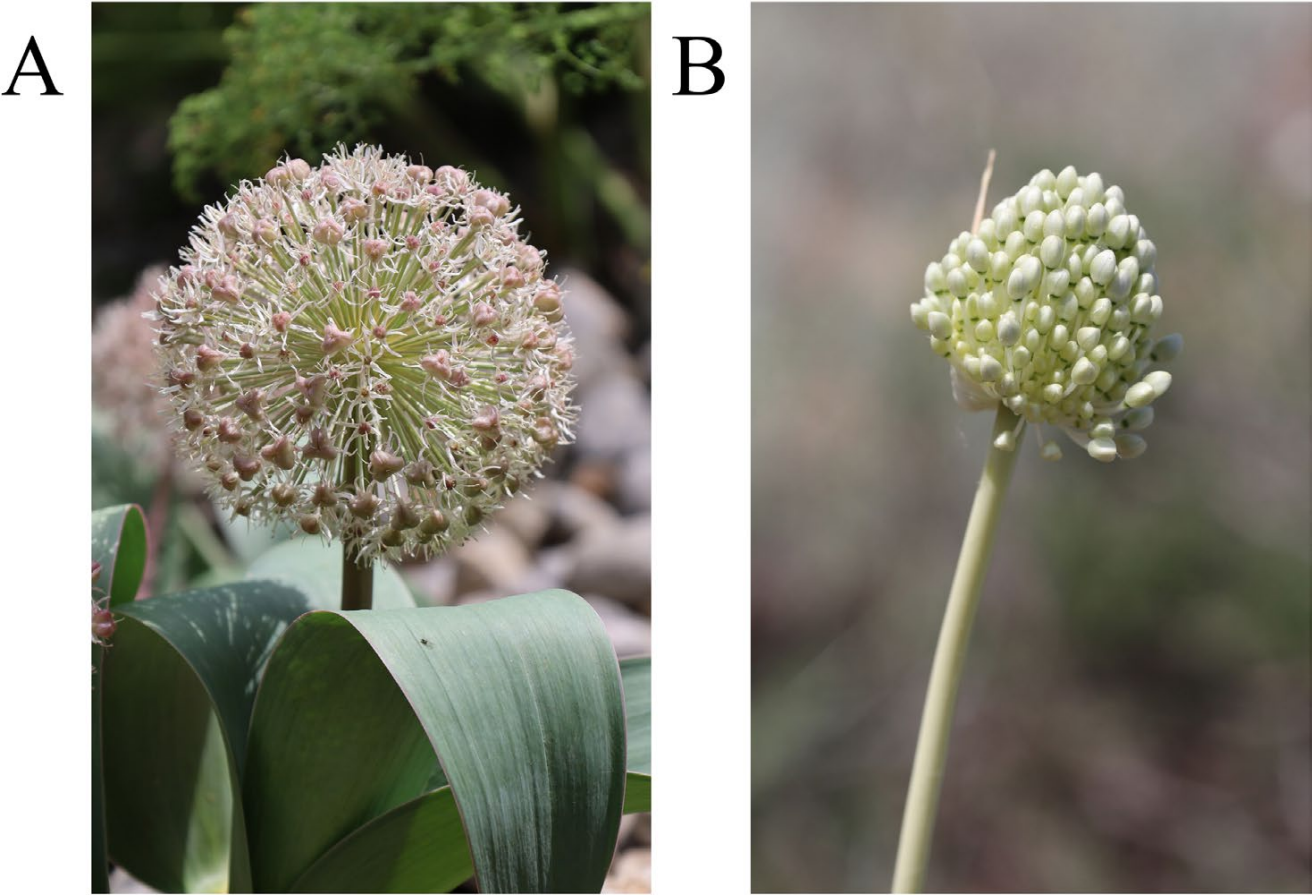
View of inflorescence of: A—*Allium karataviense*, B—*Allium turkestanicum*. Photo taken by authors of this article in 2025.

The occurrence data for *A. karataviense* and *A. turkestanicum* were obtained from the Global Biodiversity Information Facility (GBIF) (GBIF.org 2026a, 2026b). Photos of inflorescences of *A. karataviense* and *A. turkestanicum* are given in Figure 1. To reduce spatial sampling bias and local clustering, occurrence records were spatially filtered using the *spThin* package in R (Aiello‐Lammens et al. 2015). A thinning distance of 5 km was selected to match the spatial resolution of the environmental predictor layers (2.5 arc‐min), ensuring that no more than one occurrence record was retained per raster cell (Boria et al. 2014). As a result, the initial 155 occurrence points for *A. karataviense* and 43 points for *A. turkestanicum* were reduced to 51 and 25 unique locations, respectively. Final occurrence data for each species were formatted as “.csv” files for further MaxEnt modeling (Tables S1 and S2). The map of occurrence records is provided in the Supporting Information (Figure S1).

### 2.2 Collection and Analysis of Environmental Data

A total of 40 environmental variables were analyzed in this study, comprising 19 bioclimatic variables at a spatial resolution of 2.5 arc‐minutes derived from global climate and weather database (WorldClim v.2.1) (Global Climate and Weather Data 2025), two topographic variables (WorldClim v.2.1 and HWSD v.1.2) with a spatial resolution of 5 arc‐minutes each, 12 edaphic variables obtained from the Harmonized World Soil Database with spatial extent of 30 arc‐seconds (HWSD v.2.0) (FAO and International Institute for Applied Systems Analysis (IIASA) 2023), six variables from global UV‐B radiation dataset (gIUV) with a spatial resolution of 15 arc‐minutes (Beckmann et al. 2014), and the human footprint index with spatial extent of 30 arc‐seconds (Mu et al. 2022) (Table S3). All environmental predictor layers were clipped to the geographical extent of Central Asia, including Kazakhstan, Kyrgyzstan, Uzbekistan, and Tajikistan (File S1). This extent was selected because it encompasses the entire known distribution of *A. karataviense* and *A. turkestanicum* and represents the environmentally accessible area for model calibration while avoiding an unnecessarily broad background extent that could lead to model overfitting (VanDerWal et al. 2009; Barve et al. 2011). To ensure spatial consistency among datasets, all predictor variables were resampled to a common grid with a spatial resolution of 2.5 arc‐minutes using the *terra* package in R (Hijmans et al. 2026) and converted from GeoTIFF to ASCII format using package *terra* (Hijmans et al. 2026). The processed final set of environmental variables is provided in the Supporting Information (File S2).

Environmental data served as the basis for projecting the current period (1970–2000) and for future climate scenarios for 2021–2040 (2030s), 2061–2080 (2070s), and 2081–2100 (2090s). Future climate projections were based on three Shared Socioeconomic Pathways (SSPs) from the Sixth Assessment Report of the Intergovernmental Panel on Climate Change (IPCC) (Lee et al. 2023): SSP126 (sustainable development pathway), SSP245 (intermediate pathway), and SSP585 (fossil‐fueled development pathway). The bioclimatic variables were obtained from the sixth version of the Model for Interdisciplinary Research on Climate (MIROC6) in the Coupled Model Intercomparison Project Phase 6 (CMIP6) (Tatebe et al. 2019), owing to its reliability in simulating climatic conditions in Central Asia (Jiang et al. 2020; Li et al. 2020; Huang et al. 2024).

Environmental variables for MaxEnt modeling were selected through a stepwise procedure (Figure S2). Initially, all 40 candidate variables were evaluated according to their ecological relevance to the distribution and habitat requirements of *A. karataviense* and *A. turkestanicum* . Variables describing temperature, precipitation, topography, soil properties, and UV‐B radiation were considered because they are known to influence the ecology, phenology, and distribution of spring ephemeroid geophytes (Khodorova and Boitel‐Conti 2013). Subsequently, pairwise Pearson's correlation analysis was performed using the stats package in R (R Core Team 2025), and highly correlated variables (|r| > 0.7) were excluded (Saupe et al. 2012; Zhu et al. 2012). Finally, variable contribution and permutation importance were evaluated using preliminary MaxEnt models to ensure the stability of the final predictor set. The final model included variables that were both ecologically meaningful and exhibited low multicollinearity, namely temperature seasonality (BIO4), mean temperature of the wettest quarter (BIO8), annual precipitation (BIO12), precipitation seasonality (BIO15), elevation, soil properties, and UV‐B radiation, as these factors are directly associated with the growth, reproduction, and habitat requirements of the studied species (Table 1).

**TABLE 1:** Set of environmental variables used in MaxEnt modeling of *Allium karataviense* and *Allium turkestanicum*.

№	Variable	Description	Unit	Source
Bioclimatic				
1	BIO4	Temperature seasonality (standard deviation *100)	—	WorldClim v.2.1
2	BIO8	Mean temperature of wettest quarter	°C	WorldClim v.2.1
3	BIO12	Annual precipitation	mm	WorldClim v.2.1
4	BIO15	Precipitation seasonality (coefficient of variation)	—	WorldClim v.2.1
*Topographic*				
5	Elevation	Elevation	m	WorldClim v.2.1
6	Slope	Slope	%*1000	HWSD v.1.2
*UV‐B radiation*				
7	UVB2	UV‐B seasonality	J/m^2^/day	glUV
*Edaphic*				
8	CEC_EFF	Effective cation exchange capacity	cmol/kg^−1^	HWSD v.2.0
9	CEC_SOIL	Cation exchange capacity in topsoil	cmol/kg^−1^	HWSD v.2.0

### 2.3 MaxEnt Modeling

MaxEnt software version 3.4.4 (Phillips, Dudík, and Schapire 2006) was used to model the potential distribution of *A. karataviense* and *A. turkestanicum* . Model performance was evaluated using 5‐fold cross‐validation implemented in MaxEnt. Occurrence records were randomly partitioned into 5 approximately equal subsets, with each subset used once for model testing while the remaining 4 subsets were used for model training. Model performance was assessed based on the average results across the 5 cross‐validation replicates. The maximum number of iterations was set to 1500. MaxEnt was run using the default setting of 10,000 randomly distributed background points. Background points were randomly sampled across the entire study area, corresponding to the geographical extent of the environmental predictor layers. The background extent of Central Asia was selected because it represents the environmentally accessible area used for model calibration (Merow et al. 2013).

Model performance was evaluated using the area under the receiver operating characteristic curve (AUC). AUC values range from 0 to 1, with values between 0.8 and 0.9 indicating good model performance and values above 0.9 indicating excellent predictive accuracy (Swets 1988; Phillips, Anderson, and Schapire 2006; Elith et al. 2011). To assess the contribution of environmental variables, the Jackknife test, percent contribution, and permutation importance analyses were used.

In addition to AUC, model performance was evaluated using the Continuous Boyce Index (CBI) (Hirzel et al. 2006). Unlike AUC, which measures the model's ability to discriminate occurrence records from the background, the CBI evaluates the consistency between predicted habitat suitability and the observed distribution of presence records. CBI values range from −1 to 1, where positive values close to 1 indicate high predictive performance, values around 0 indicate performance no better than random, and negative values indicate poor model predictions. CBI values were calculated using the R package *ecospat* (Di Cola et al. 2017).

Habitat suitability thresholds were determined using the Continuous Boyce Index (CBI) and the Boyce predicted‐to‐expected (P/E) ratio curve (Hirzel et al. 2006). The first threshold separating unsuitable and suitable habitats was defined as the habitat suitability value at which the Boyce P/E ratio approached 1, indicating that model predictions became better than random expectations. Additional ecological thresholds were identified by applying segmented regression to the Boyce P/E response curve (Yu et al. 2024). A linear model was fitted to the relationship between habitat suitability and the Boyce P/E ratio, and breakpoints were estimated using segmented regression with the R package *segmented* (Muggeo 2008). The resulting species‐specific thresholds were used to classify habitat suitability into four categories in QGIS v.3.40.8 (QGIS.org 2026): unsuitable (white), low suitability (yellow), moderate suitability (green), and high suitability (red) categories. Raster layers were subsequently reclassified according to these thresholds, and the area of each suitability class was calculated in square kilometers using PyQGIS.

### 2.4 Centroid Migration Analysis

Centroids of potential habitats were calculated using the R package *terra* (Hijmans et al. 2026). Centroids represent the area‐weighted geographic centers of suitable habitats. For this analysis, areas classified as having moderate and high suitability were combined into a single binary raster layer and subsequently converted to polygons for centroid extraction. Centroids were generated for three climate scenarios over four time periods, yielding a total of 10 centroids per species. Migration distances between centroids across different times were calculated using the R package *terra* (Hijmans et al. 2026). Finally, the geographic coordinates of the centroids were extracted and visualized on a map using QGIS.

### 2.5 Analysis of the Intersection Between Existing Protected Areas and Suitable Habitats, With Identification of Potential IPAs

Spatial data on Specially Protected Natural Territories (SPNTs) in Kazakhstan were obtained from the official database (www.oopt.kz) (Specially Protected Natural Territories (SPNTs) of Kazakhstan 2026), while data on protected areas in neighboring countries were retrieved from the Protected Planet website (UNEP‐WCMC and IUCN 2026). The compiled dataset included National and Regional Reserves, State Nature Reserves, State National Nature Parks, and other effective area‐based conservation measures (File S3).

For each analyzed time period, areas with high suitability were merged into a single spatial layer and overlaid on the boundaries of SPNTs. The intersection between suitable habitats and protected areas was calculated to evaluate how well these species are represented within existing conservation networks and to identify IPAs for future conservation efforts.

The R scripts used for environmental data processing, variable selection, model evaluation, and other supporting datasets are provided in the Supporting Information (File S4).

## 3 Results

### 3.1 MaxEnt Model Reliability Assessment

**FIGURE 2:**
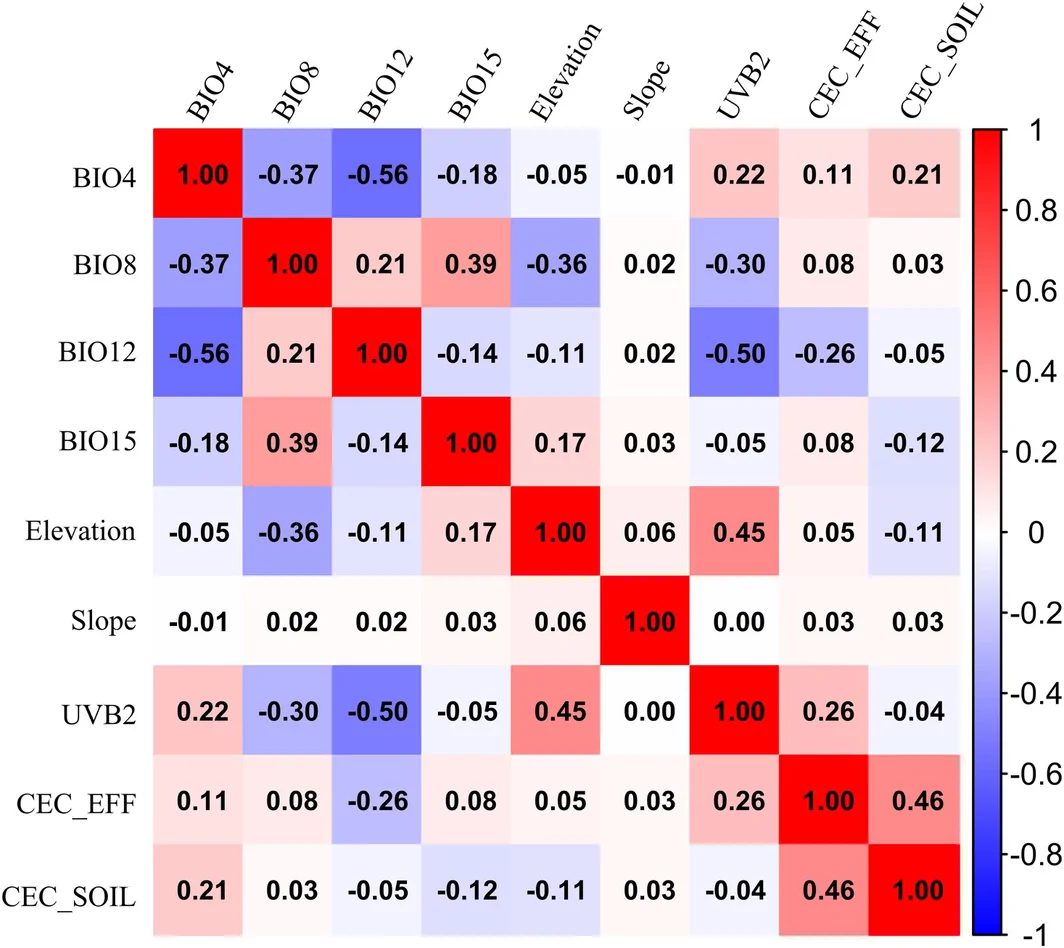
Pairwise Pearson's correlations of nine environmental variables used for MaxEnt modeling. Red indicates positive correlations, while blue indicates negative. BIO4—Temperature Seasonality; BIO8—Mean Temperature of Wettest Quarter; BIO12—Annual Precipitation; BIO15—Precipitation Seasonality (Coefficient of Variation); UVB2—UV‐B Seasonality; CEC_EFF—Effective cation exchange capacity; CEC_SOIL—Cation exchange capacity in topsoil.

Initially, 40 environmental variables were analyzed using pairwise correlations to evaluate multicollinearity prior to MaxEnt modeling. After that, nine variables with correlation coefficients not exceeding |r| = 0.7 were selected (Figure 2). Correlation coefficients ranged from −0.56 to 0.45. The strongest negative correlation was observed between BIO12 and BIO4 (r = −0.56), followed by BIO12 and UVB2 (r = −0.50). The strongest positive correlation was found between CEC**_**SOIL and CEC**_**EFF (*r* = 0.46), followed by Elevation and UVB2 (*r* = 0.45). Other variable pairs exhibited weak to moderate correlations (|r| < 0.4), indicating overall low multicollinearity among the selected variables, which were subsequently retained for further modeling.

The MaxEnt models were constructed to predict suitable habitats for *A. karataviense* and *A. turkestanicum* using nine environmental variables across three climate change scenarios (ssp126, ssp245, ssp585) and four time periods (Current, 2030s, 2070s, 2090s). In total, 10 separate models for each species were constructed to represent the different scenario–time combinations.

**FIGURE 3:**
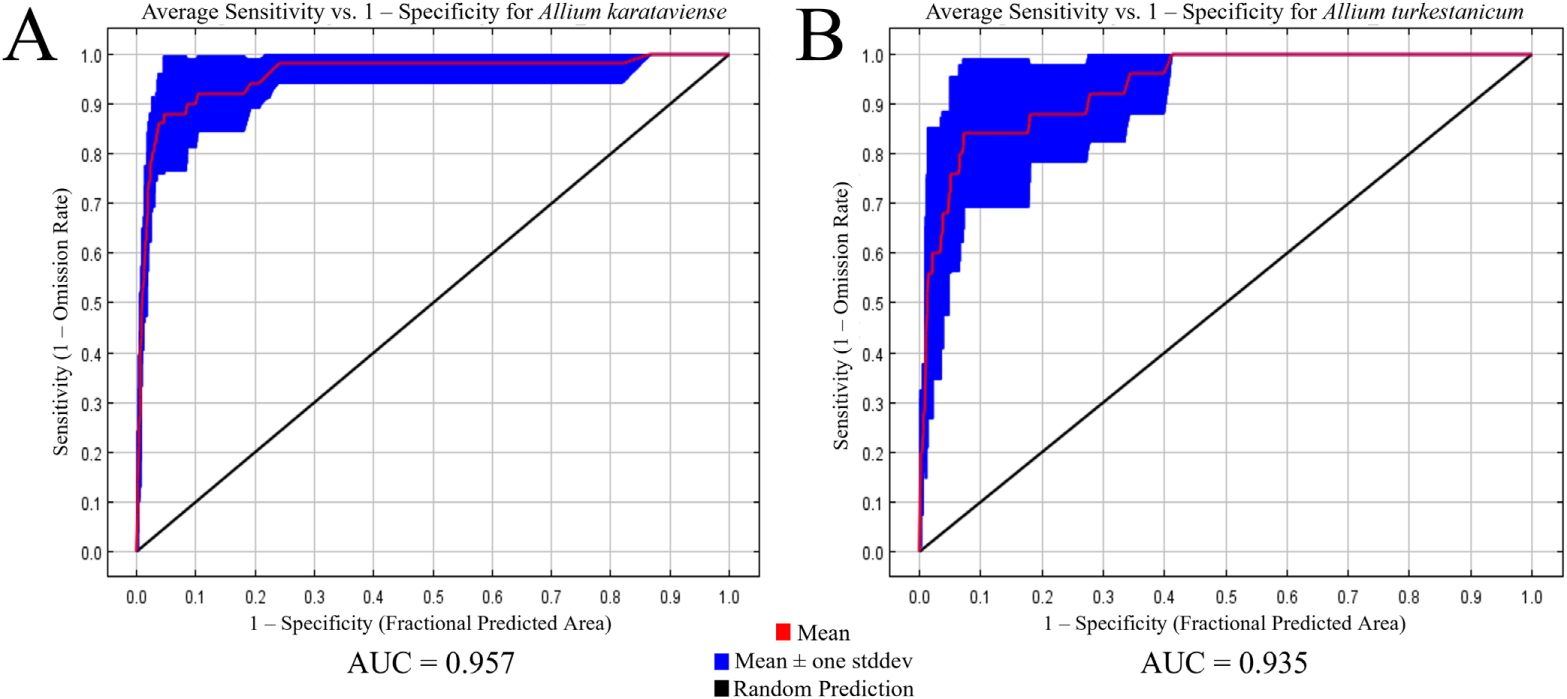
Receiver operating characteristic (ROC) curve and AUC values of the MaxEnt models for: A—*Allium karataviense*, B—*Allium turkestanicum*.

Models of *A. karataviense* demonstrated an average AUC value of 0.957 for selected environmental variables (Figure 3A), while models of *A. turkestanicum* had an average AUC value equal to 0.935 (Figure 3B).

The CBI values were 0.938 for *A. karataviense* and 0.878 for *A. turkestanicum* . Species‐specific habitat suitability thresholds derived from the Boyce P/E curve and segmented regression were used to classify habitat suitability into four ecological categories. For *A. karataviense*, the thresholds were identified at HS = 0.221, 0.599, and 0.791, defining unsuitable (< 0.221), low (0.221–0.599), moderate (0.599–0.791), and high suitability (≥ 0.791) habitats. For *A. turkestanicum* , the thresholds were identified at HS = 0.266, 0.323, and 0.666, defining unsuitable (< 0.266), low (0.266–0.323), moderate (0.323–0.666), and high suitability (≥ 0.666) habitats. The Boyce P/E curves, segmented regression models, and corresponding statistical results are provided in Figure S3 and Table S4.

For *A. karataviense*, the four most important environmental variables were Slope (39.7%), Elevation (21.2%), UVB2 (17.9%), and BIO15 (8.6%). BIO4 contributed an additional 7.2%, whereas the remaining variables each contributed less than 2%. According to permutation importance, Elevation (25.6%) had the highest influence, followed by Slope (21.3%), UVB2 (20.6%), BIO8 (14.0%), and BIO15 (11.9%), while the other variables showed relatively low importance (Table 2).

**TABLE 2:** Contribution rates of environmental variables for *Allium karataviense* and *Allium turkestanicum*.

№	Variable	*A. karataviense*		Variable	*A. turkestanicum*	
		Percent contribution, %	Permutation importance, %		Percent contribution, %	Permutation importance, %
1	Slope	39.7	21.3	Elevation	39.6	51
2	Elevation	21.2	25.6	UVB2	17.5	26.1
3	UVB2	17.9	20.6	Slope	11.4	1
4	BIO15	8.6	11.9	BIO8	10.4	7.5
5	BIO4	7.2	4.7	CEC_SOIL	7.5	6.5
6	BIO12	1.9	0.6	BIO15	5.3	2.9
7	BIO8	1.8	14	CEC_EFF	4.1	0
8	CEC_EFF	1.6	0	BIO12	3.3	2.1
9	CEC_SOIL	0.2	1.3	BIO4	0.9	2.8

For *A. turkestanicum* , Elevation (39.6%), UVB2 (17.5%), Slope (11.4%), and BIO8 (10.4%) were the main contributing variables. CEC_SOIL (7.5%), BIO15 (5.3%), and CEC_EFF (4.1%) contributed additionally. In terms of permutation importance, Elevation (51.0%) was by far the most influential predictor, followed by UVB2 (26.1%) and BIO8 (7.5%) (Table 2).

**FIGURE 4:**
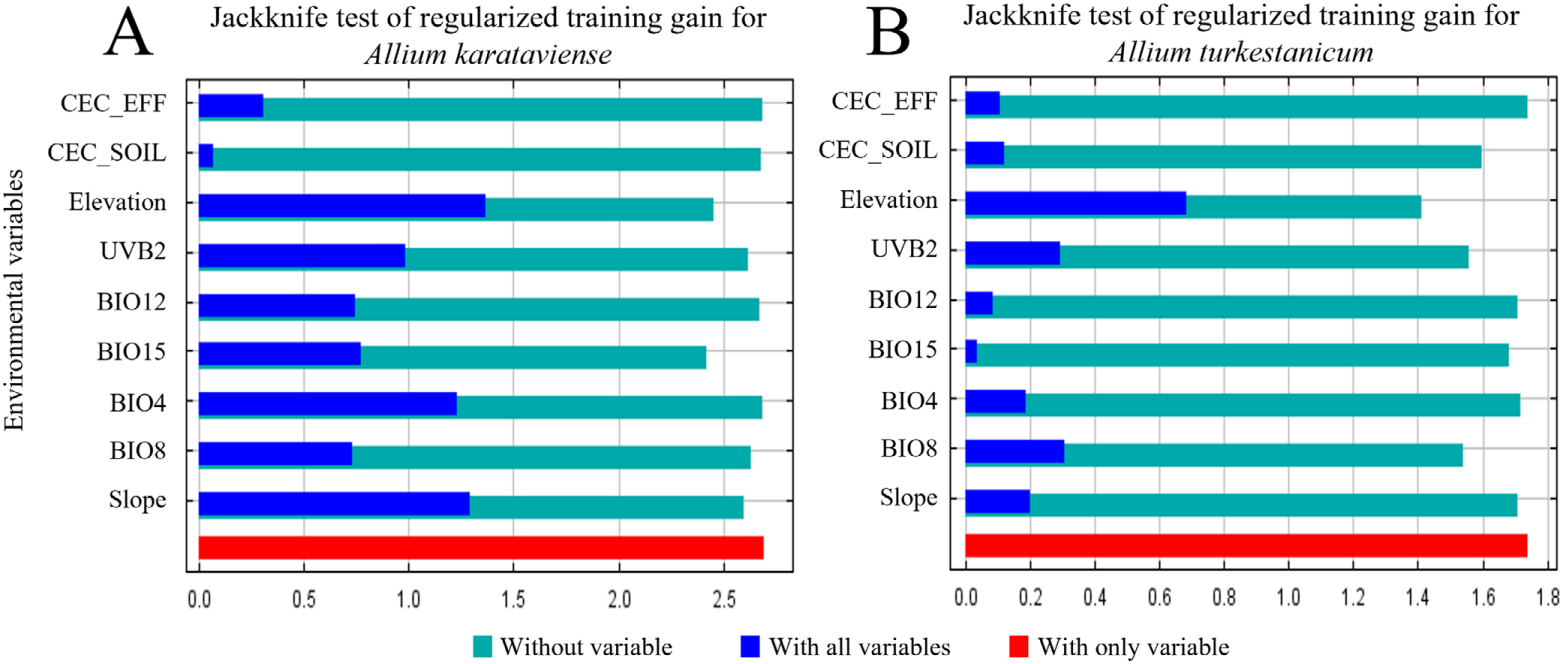
Jackknife test of environmental variables importance for: A—*Allium karataviense*, B—*Allium turkestanicum*.

The contribution of environmental variables to the geographical distribution of *A. karataviense* and *A. turkestanicum* was evaluated using the Jackknife test based on regularized training gain (Figure 4). For *A. karataviense*, Slope was the most informative predictor, followed by Elevation, BIO4, and UVB2. In contrast, Elevation was the key variable determining the distribution of *A. turkestanicum* , followed by UVB2 and BIO8 (Figure 4).

**FIGURE 5:**
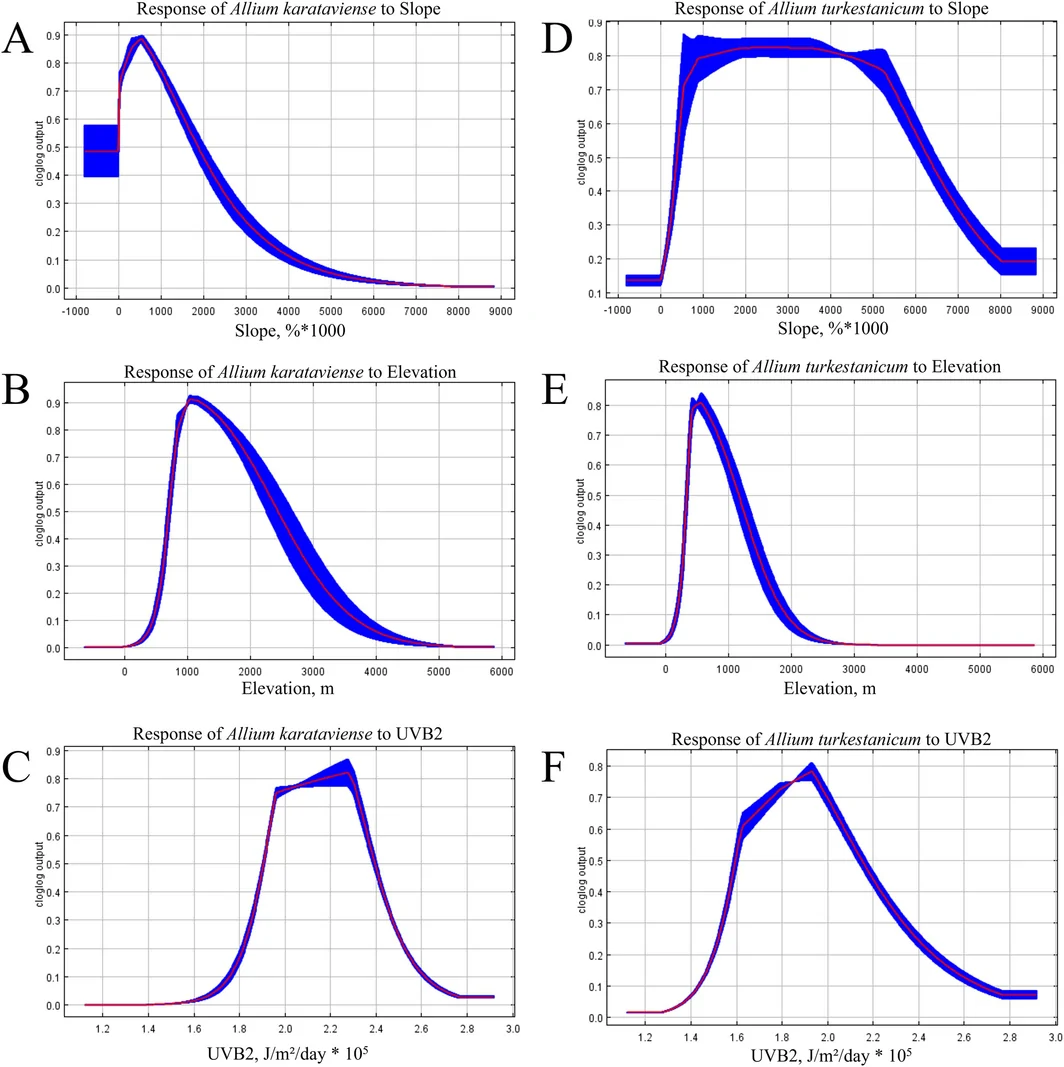
Response curves of the main environmental variables of *Allium karataviense*: A—Slope, B—Elevation, C—UVB2, and *Allium turkestanicum*: D—Slope, E—Elevation, F—UVB2.

Response curves revealed distinct ecological responses of the two *Allium* species to the most influential environmental variables (Figure 5).

For *A. karataviense*, habitat suitability was highest at low slope values and decreased continuously with increasing values (Figure 5A). In relation to elevation, suitability increased rapidly to a maximum at approximately 1000 m and then gradually declined at higher elevations (Figure 5B). The response to UVB2 showed increasing values around 1.8 × 10^5^ J/m^2^/day, reaching maximum suitability at approximately 2.3 × 10^5^ J/m^2^/day (Figure 5C). For BIO4, the probability of occurrence increased sharply at temperature seasonality values of approximately 900–1100 and remained close to its maximum at higher values, indicating a preference for areas with moderate and high temperature seasonality (Figure S4). For BIO8, habitat suitability was highest at temperatures of approximately 5°C–10°C and declined toward both lower and higher temperatures, indicating an intermediate optimum for the temperature of the wettest quarter (Figure S4). BIO12 response curve (Figure S4) showed the probability of occurrence increased rapidly with increasing annual precipitation and reached a plateau at approximately 700–900 mm. Response to BIO15 showed highest suitability at intermediate values (approximately 55), whereas suitability declined under both low and high precipitation seasonality (Figure S4).

In contrast, *A. turkestanicum* exhibited high habitat suitability across a broad range of slope values (approximately 1000–4000), after which suitability declined markedly at steeper slopes. The elevation response indicated an optimum at relatively low elevations (approximately 500 m), followed by a rapid decrease with increasing altitude. Similarly, the response to UVB2 was unimodal, with the highest suitability at intermediate values (approximately 1.9 × 10^5^ J/m^2^/day) and progressively lower suitability beyond this optimum. The response to BIO8 was more complex and showed a bimodal pattern. Suitability remained very high at low BIO8 values, decreased sharply around −5°C to 0°C, then increased again to around 10°C–12°C. Thereafter, suitability declined rapidly, reaching very low values at BIO8 values above approximately 20 (Figure S5). The response to BIO12 showed an initial sharp increase in suitability, followed by a maximum at around 400 mm. Suitability subsequently declined gradually with increasing precipitation, reaching low values at around 1000 mm (Figure S5). Habitat suitability increased gradually with BIO15 and reached a maximum around 55, after which it decreased steadily (Figure S5). The response to BIO4 was generally unimodal. Suitability increased from low values and reached a maximum around 1100–1200, followed by a pronounced decline at 1350–1400. At higher BIO4 values (1550–1600), suitability increased again (Figure S5).

### 3.2 Analysis of Predicted Current and Future Suitable Distribution Areas

The predicted suitable distribution areas of *A. karataviense* and *A. turkestanicum* varied across climate scenarios and time periods, showing both quantitative and proportional changes (Table 3).

**TABLE 3:** Predicted current and future suitable distribution areas of *Allium karataviense* and *Allium turkestanicum*.

Species	Period	Predicted suitable distribution area, km^2^			
		Low	Moderate	High	*Total*
*A. karataviense*	Current	97,427	24,117	28,971	150,515
	ssp126				
	2030s	80,194	22,699	28,792	131,685
	2070s	90,677	25,641	30,225	146,543
	2090s	87,929	23,132	30,166	141,227
	ssp245				
	2030s	99,548	22,400	28,762	150,710
	2070s	95,904	27,537	29,792	153,233
	2090s	100,981	25,924	29,001	155,906
	ssp585				
	2030s	88,646	23,207	29,538	141,391
	2070s	96,889	25,626	29,927	152,442
	2090s	101,34	26,94	32,346	160,626
*A. turkestanicum*	Current	61,989	198,902	120,500	381,391
	ssp126				
	2030s	80,657	246,914	112,316	439,887
	2070s	64,513	241,089	126,742	432,344
	2090s	71,622	275,019	117,259	463,900
	ssp245				
	2030s	64,827	289,773	121,44	476,04
	2070s	57,868	194,063	132,999	384,930
	2090s	61,049	223,557	120,082	404,688
	ssp585				
	2030s	81,314	241,776	131,371	454,461
	2070s	49,833	206,324	114,780	370,937
	2090s	54,209	234,115	113,227	401,551

**FIGURE 6:**
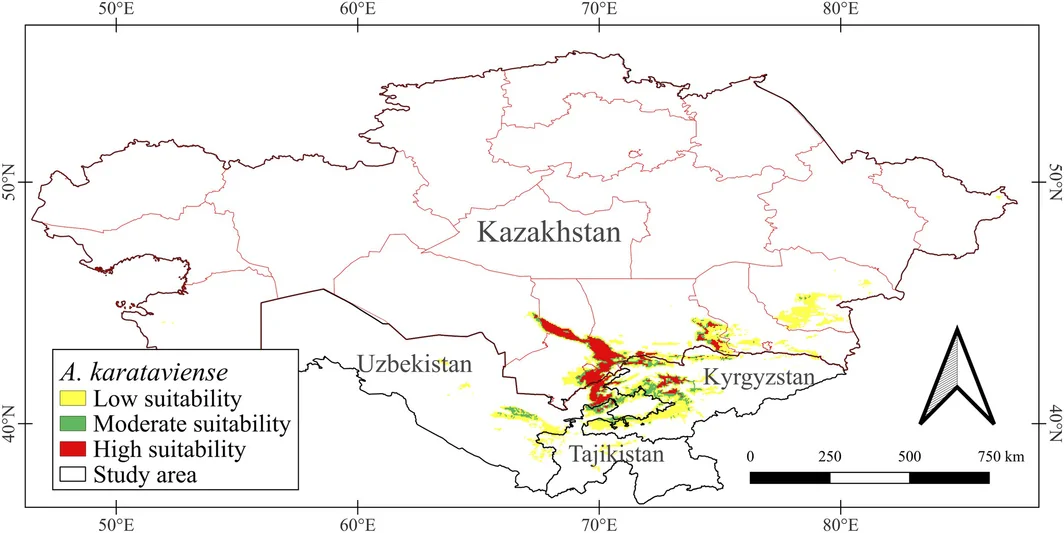
Current predicted habitat of *Allium karataviense*.

For *A. karataviense*, the current total suitable area is 150,515 km^2^, of which 97,427 km^2^ (64.7%) is classified as low suitability, 24,117 km^2^ (16.0%) as moderate suitability, and 28,971 km^2^ (19.2%) as high suitability (Table 3). The species is predominantly distributed across southern and southeastern Kazakhstan, western Kyrgyzstan, and parts of Uzbekistan and Tajikistan (Figure 6).

**FIGURE 7:**
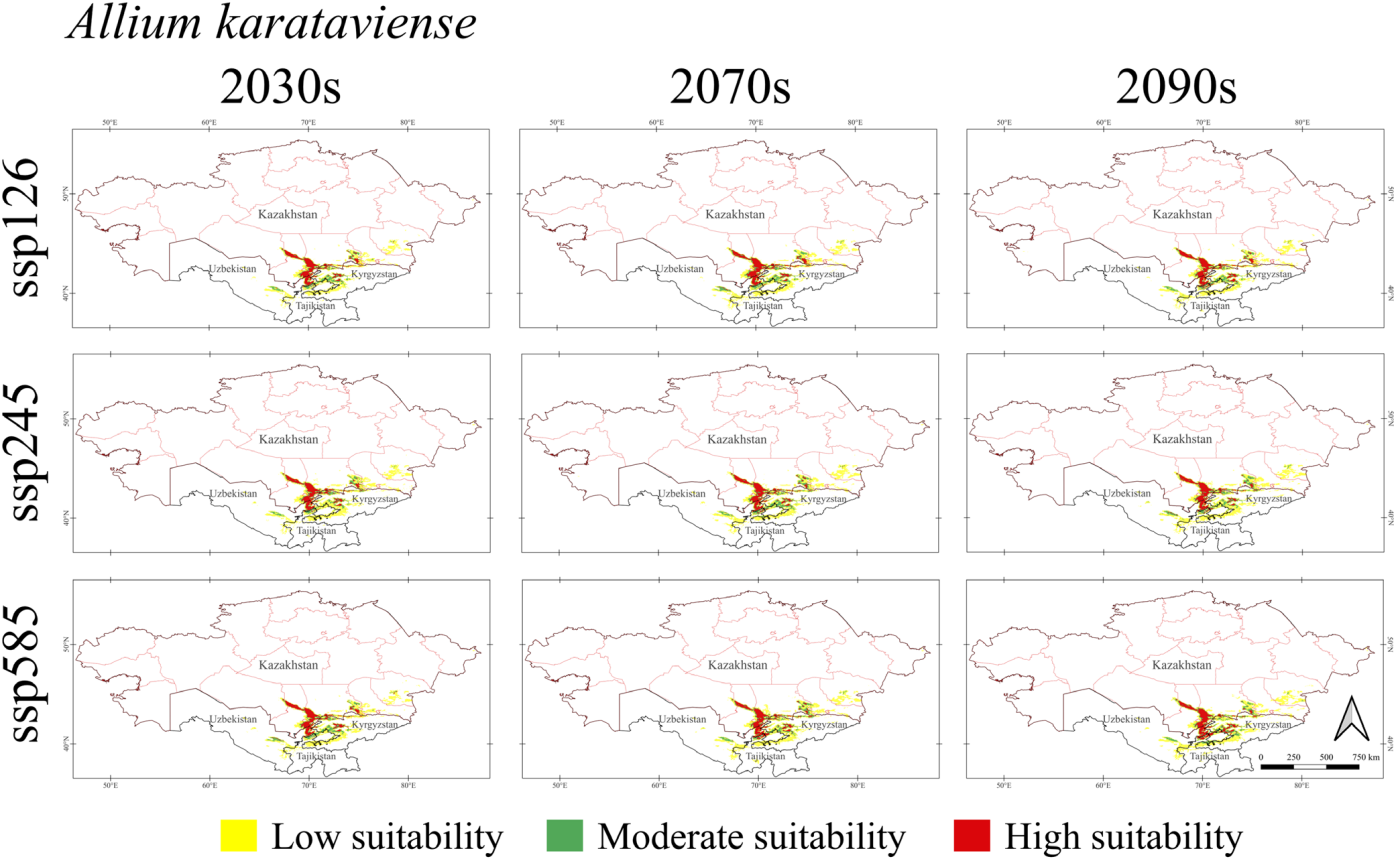
Future predicted suitable areas for *Allium karataviense*.

Under the ssp126 scenario, the total suitable area decreases to 131,685 km^2^ in the 2030s, with low suitability accounting for approximately 60.9% (80,194 km^2^) and high suitability representing about 21.9% (28,792 km^2^). By the 2070s, the total area slightly increases to 146,543 km^2^, while in the 2090s it decreases to 141,227 km^2^ (Table 3, Figure 7).

Under ssp245, the total suitable area reaches 150,710 km^2^ in the 2030s and increases slightly to 153,233 km^2^ in the 2070s and 155,906 km^2^ in the 2090s. High suitability remains relatively stable, ranging from 28,762 km^2^ (19.1%) in the 2030s to 29,792 km^2^ (19.4%) in the 2070s and 29,001 km^2^ (18.6%) in the 2090s. Moderate and high suitability together account for 33%–36% of the total area, indicating relatively stable distributions of the more suitable habitats (Table 3, Figure 7).

The most pronounced increase in total suitable area occurs under ssp585, where the area reaches 141,391 km^2^ in the 2030s, increases to 152,442 km^2^ in the 2070s, and peaks at 160,626 km^2^ in the 2090s. High suitability increases from 29,538 km^2^ (20.9%) in the 2030s to 32,346 km^2^ (20.1%) by the 2090s, while low suitability remains the dominant class throughout the period. Overall, the projections indicate a moderate expansion of suitable habitats under ssp585, particularly toward the southeastern parts of Kazakhstan and adjacent areas of Kyrgyzstan (Table 3, Figure 7).

**FIGURE 8:**
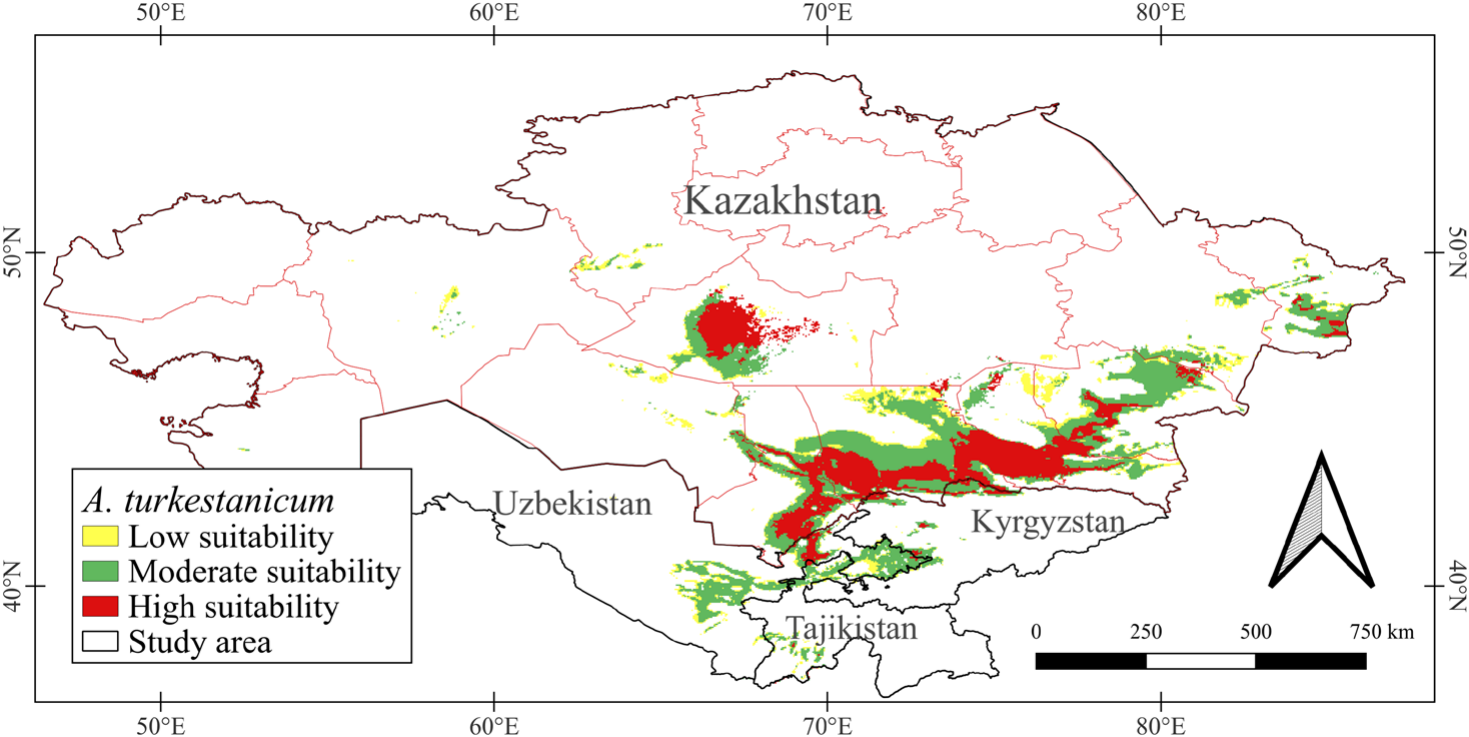
Current predicted habitat of *Allium turkestanicum*.

In contrast, *A. turkestanicum* shows a current suitable area of 381,391 km^2^, including 61,989 km^2^ (16.2%) of low suitability, 198,902 km^2^ (52.1%) of moderate suitability, and 120,500 km^2^ (31.6%) of high suitability (Table 3). The species has a wide distribution range comprising southern and southeastern Kazakhstan, parts of central Kazakhstan, western Kyrgyzstan, eastern Uzbekistan, and northern parts of Tajikistan (Figure 8).

**FIGURE 9:**
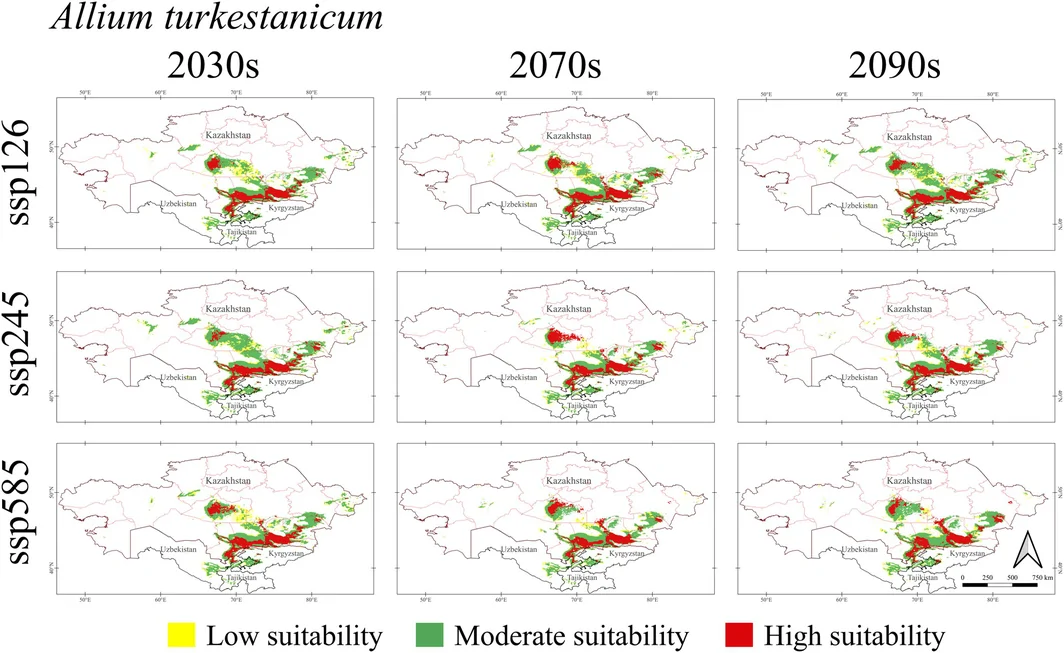
Future predicted suitable areas for *Allium turkestanicum*.

Under ssp126, the total suitable area increases to 439,887 km^2^ in the 2030s, with moderate and high suitability accounting for 56.1% (246,914 km^2^) and 25.5% (112,316 km^2^), respectively. The total area slightly decreases to 432,344 km^2^ in the 2070s before increasing to 463,900 km^2^ in the 2090s. By the 2090s, moderate suitability reaches 275,019 km^2^ (59.3%), while high suitability accounts for 117,259 km^2^ (25.3%). Spatial changes are mainly observed in central and southeastern Kazakhstan (Table 3, Figure 9).

Under ssp245, suitable area increases to 476,040 km^2^ in the 2030s, decreases to 384,930 km^2^ in the 2070s, and then increases to 404,688 km^2^ in the 2090s. Moderate suitability accounts for 289,773 km^2^ (60.9%) in the 2030s and decreases to 223,557 km^2^ (55.2%) by the 2090s, while high suitability remains relatively stable (121,440 km^2^ to 120,082 km^2^). Changes are mainly concentrated in central Kazakhstan and the southeastern part of the species' range (Table 3, Figure 9).

Under ssp585, the total suitable area reaches 454,461 km^2^ in the 2030s, decreases to 370,937 km^2^ in the 2070s, and increases to 401,551 km^2^ in the 2090s. Moderate suitability remains dominant, accounting for 241,776 km^2^ (53.2%) in the 2030s and 234,115 km^2^ (58.3%) in the 2090s, while high suitability decreases from 131,371 km^2^ (28.9%) to 113,227 km^2^ (28.2%). Overall, suitable habitat shows substantial temporal fluctuations, with the largest area occurring under ssp245 in the 2030s. Changes are concentrated mainly in central and southeastern Kazakhstan, with additional shifts in adjacent areas of Kyrgyzstan and Uzbekistan (Table 3, Figure 9).

### 3.3 Migration of Centroids of Future Suitable Habitats

**FIGURE 10:**
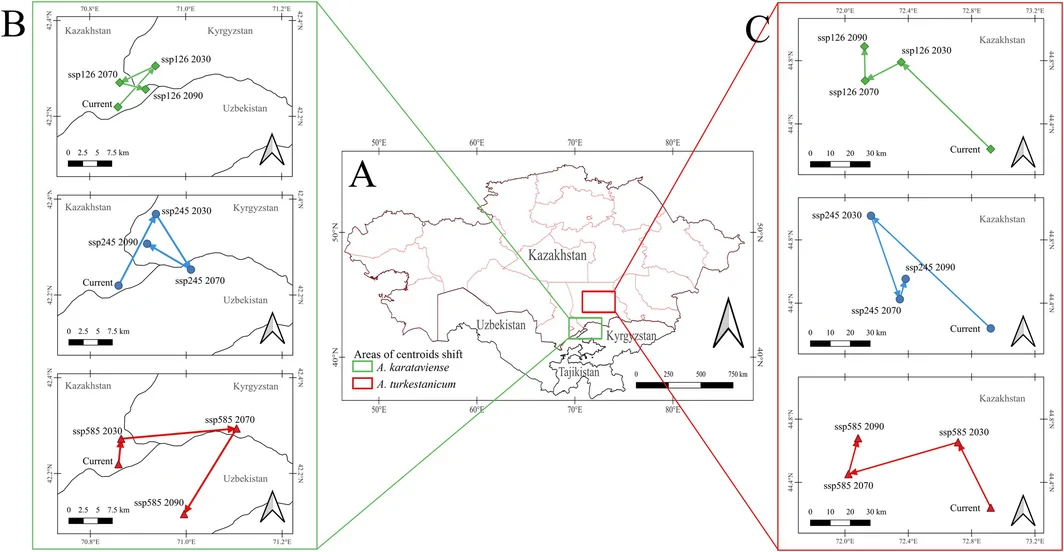
Centroid migration maps: A—general view, B—centroids of *Allium karataviense*, C—centroids of *Allium turkestanicum*. Green points indicate ssp126, blue points indicate ssp245, and red points indicate ssp585.

The analysis of centroid migration indicates that both *A. karataviense* and *A. turkestanicum* are expected to undergo spatial redistribution under future climate scenarios, with differences in migration magnitude and direction among scenarios and time periods (Figure 10, Table 4).

**TABLE 4:** Centroid migration distances of *Allium karataviense* and *Allium turkestanicum* under future climate scenarios.

Species	Period	Longitude (°E)	Latitude (°N)	Direction	Migration distance, km
*A. karataviense*	Current	70.86	42.22	—	—
	ssp126				
	2030s	70.94	42.31	Current→2030s	11.530
	2070s	70.86	42.27	2030s→2070s	7.274
	2090s	70.92	42.26	2070s→2090s	4.707
	ssp245				
	2030s	70.94	42.37	Current→2030s	17.827
	2070s	71.01	42.25	2030s→2070s	14.242
	2090s	70.92	42.31	2070s→2090s	9.639
	ssp585				
	2030s	70.86	42.27	Current→2030s	5.927
	2070s	71.11	42.29	2030s→2070s	19.957
	2090s	71.00	42.12	2070s→2090s	21.824
*A. turkestanicum*	Current	72.92	44.24	—	—
	ssp126				
	2030s	72.36	44.79	Current→2030s	75.916
	2070s	72.13	44.67	2030s→2070s	22.216
	2090s	72.12	44.89	2070s→2090s	24.082
	ssp245				
	2030s	72.16	44.95	Current→2030s	99.382
	2070s	72.35	44.42	2030s→2070s	60.452
	2090s	72.38	44.55	2070s→2090s	14.623
	ssp585				
	2030s	72.71	44.65	Current→2030s	48.904
	2070s	72.02	44.45	2030s→2070s	59.320
	2090s	72.08	44.68	2070s→2090s	25.690

For *A. karataviense*, centroid movements are generally limited, indicating relatively stable spatial dynamics (Table 4, Figure 10B). There is also a tendency to move eastward across all scenarios. At present, the centroid position is at 70.86**°** E, 42.22**°** N. Under the ssp126 scenario, the species shows a moderate shift of 11.530 km from the current period to the 2030s, followed by minimal movement toward the 2070s (7.274 km) and a slight southward shift by the 2090s (4.707 km), reflected in decreasing latitude values. Under ssp245, the initial shift is substantial (17.827 km) toward the 2030s, followed by further displacement in the 2070s (14.242 km) and subsequent stabilization in the 2090s (9.639 km). In the high‐emission ssp585 scenario, centroid shifts remain minor in the 2030s (5.927 km) but increase to 19.527 km in the 2070s and 21.824 km by the 2090s, indicating delayed but more noticeable movement (Table 4).

In contrast, *A. turkestanicum* demonstrates substantially greater centroid displacement across all scenarios, indicating higher sensitivity to environmental changes (Table 4, Figure 10C). Centroid movement shows a northwestward trend. Currently, the centroid is located at 72.92° E longitude and 44.24° N latitude. Under ssp126, the species shifts by 75.916 km toward the 2030s, followed by continued movement in the 2070s (22.216 km) and 2090s (24.082 km), with a general tendency toward higher latitudes. Under ssp245, even larger shifts are observed, with an initial displacement of 99.382 km, followed by 60.452 km and 14.623 km in subsequent periods, suggesting persistent redistribution. Under ssp585, centroid shifts reach 48.904 km toward the 2030s and 59.320 km toward the 2070s and remain high (25.690 km) toward the 2090s. These results indicate strong and continuous movement, particularly under high‐emission conditions (Table 4).

### 3.4 Analysis of the Intersection Between Existing Protected Areas and Suitable Habitats

**FIGURE 11:**
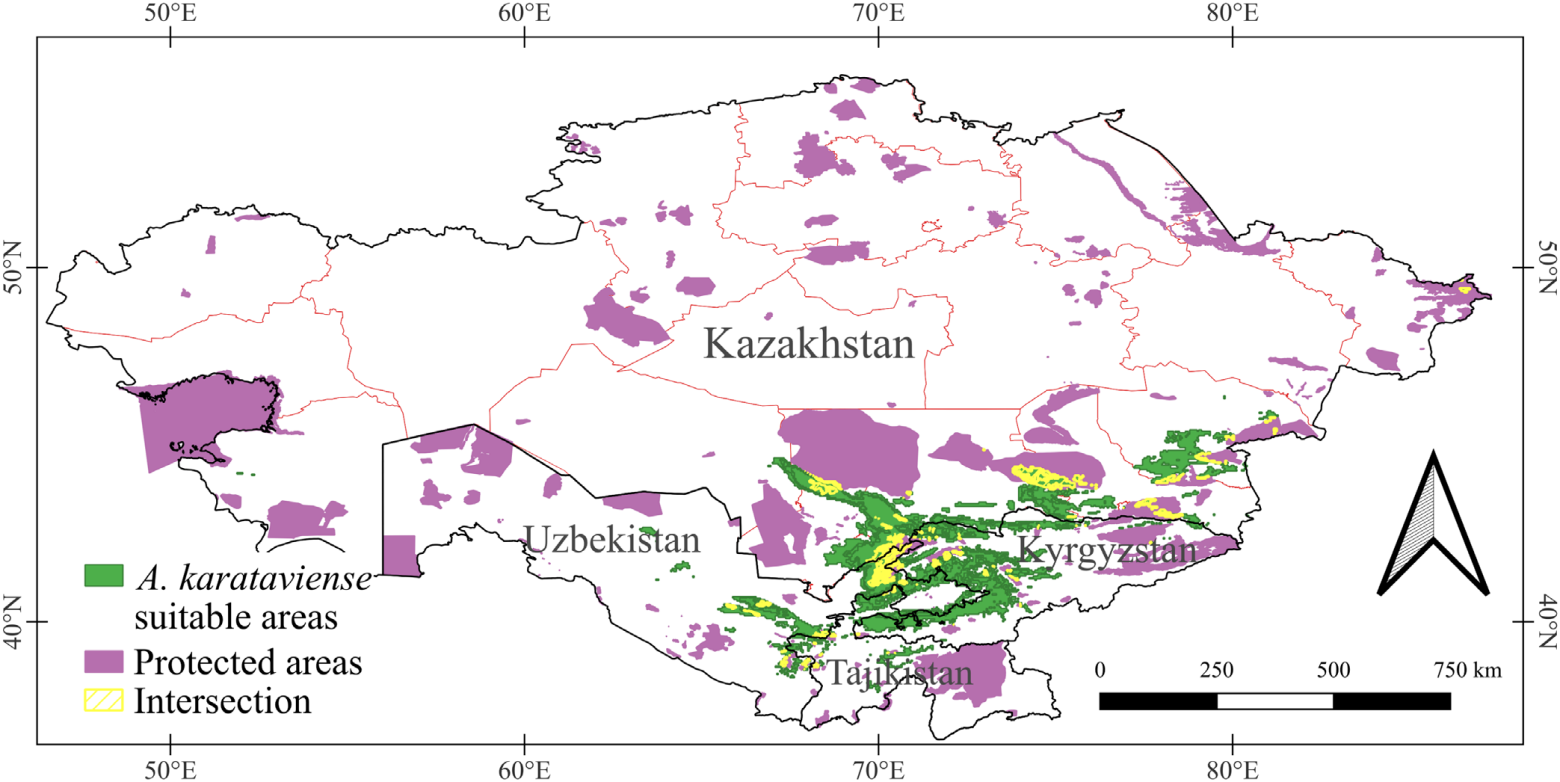
Overlap map between specially protected natural territories (SPNTs) and the highly suitable areas of *Allium karataviense*.

**FIGURE 12:**
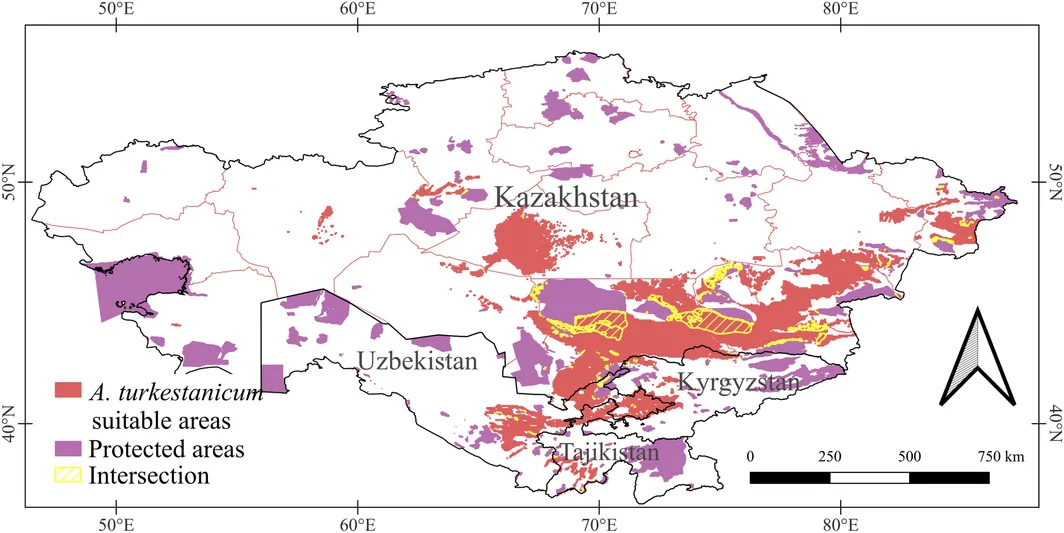
Overlap map between specially protected natural territories (SPNTs) and the highly suitable areas of *Allium turkestanicum*.

The spatial overlap between suitable habitats and existing protected areas revealed differences in the extent to which the predicted distributions of *A. karataviense* and *A. turkestanicum* are currently represented within the protected‐area network (Table 5, Figures 11 and 12).

**TABLE 5:** Spatial intersection between suitable habitats and specially protected natural territories (SPNTs) for *Allium karataviense* and *Allium turkestanicum* across Central Asia.

*A. karataviense*				
№	Country	Number of SPNTs	Area of intersection, km^2^	% of suitable area
1	Kazakhstan	18	11,214	7.45
2	Uzbekistan	11	4696	3.12
3	Kyrgyzstan	16	1287	0.85
4	Tajikistan	6	333	0.22
Total		51	17,529	11.65
*A. turkestanicum*				
1	Kazakhstan	35	47,926	12.57
2	Uzbekistan	12	541	0.14
3	Tajikistan	5	62	0.02
4	Kyrgyzstan	4	22	0.01
Total		56	48,551	12.73

For *A. karataviense*, the largest share of intersection occurs in Kazakhstan, with 11,214 km^2^ (7.45% of the highly suitable area). Further significant areas are observed in Uzbekistan (4696 km^2^, 3.12%) and Kyrgyzstan (1287 km^2^, 0.85%), while Tajikistan shows minimal overlap (333 km^2^, 0.22%). The largest individual protected areas for *A. karataviense* include Zhusandala State Reserved Zone, Kazakhstan (5332 km^2^), Ugam‐Chatkal State National Nature Park, Uzbekistan (3314 km^2^), and the Sayram‐Ugam State National Nature Park, Kazakhstan (1120 km^2^). These areas represent key conservation hotspots for the species (Table 5, Figure 11). In total, 51 SPNTs cover 17,529 km^2^, accounting for 11.65% of the species' suitable habitats.

For *A. turkestanicum* , the overlap is strongly concentrated in Kazakhstan, where 47,926 km^2^ (12.57%) of suitable habitat falls within protected areas. In contrast, Uzbekistan (541 km^2^; 0.14%), Tajikistan (62 km^2^; 0.02%), and Kyrgyzstan (22 km^2^; 0.01%) are protected only marginally. The largest protected areas for *A. turkestanicum* are all located in Kazakhstan and include South Kazakhstan State Reserved Zone (18,513 km^2^), Zhusandala State Reserved Zone (17,529 km^2^), and Karoi State Nature Sanctuary (2377 km^2^) (Table 5, Figure 12). Overall, 56 protected areas cover 48,551 km^2^, corresponding to 12.73% of the highly suitable habitats.

## 4 Discussion

The MaxEnt models developed for *A. karataviense* and *A. turkestanicum* demonstrated high discriminatory performance, as indicated by AUC values of 0.957 and 0.935, respectively (Figure 2). These values indicate a high ability of the models to distinguish presence locations from the background environment. This interpretation is further supported by the high CBI values (0.938 and 0.878, for *A. karataviense* and *A. turkestanicum* , respectively), which indicate strong agreement between predicted habitat suitability and observed species occurrences (Hirzel et al. 2006). The use of both evaluation metrics provides greater confidence in the predictive performance of the developed models. The species distribution data and environmental variable data used in the modeling can effectively predict the potentially suitable distribution area of both species in Central Asia (Pearce and Ferrier 2000; Duan et al. 2025). Moreover, using species‐specific ecological thresholds derived from the Boyce P/E curve and segmented regression provides a biologically meaningful alternative to arbitrary or fixed threshold values used in species distribution modeling. Unlike conventional threshold selection methods, this approach identifies changes in habitat suitability based on the relationship between predicted suitability and species occurrence, allowing habitat classes to reflect species‐specific ecological responses (Liu et al. 2013; Yu et al. 2024). Consequently, the resulting habitat suitability categories are more ecologically interpretable and may improve the identification of priority areas for conservation and management.

The analysis of variable importance revealed both common and species‐specific environmental drivers of habitat suitability (Table 2). Interestingly, Elevation, Slope, and UVB2 consistently ranked among the three most important environmental variables for both species, indicating that topographic conditions and solar radiation represent common environmental factors influencing their distributions. For *A. karataviense*, Slope had the highest percentage contribution (39.7%), followed by Elevation (21.2%) and UVB2 (17.9%). This strong influence of topographic variables is consistent with the species' association with mountainous environments, where terrain characteristics, elevation, and radiation conditions may act as important ecological drivers (Fagre et al. 2003; Turunen and Latola 2005; Wang et al. 2025). For *A. turkestanicum* , Elevation was the dominant predictor, accounting for 39.6% of the contribution, followed by UVB2 (17.5%) and Slope (11.4%). The strong influence of Elevation, together with the lower contribution of Slope, suggests that altitude is a particularly important factor defining the distribution of *A. turkestanicum* , while UVB2 represents an additional important environmental constraint. Other variables, including BIO8, BIO15, BIO4, and soil‐related predictors, made smaller but measurable contributions to model performance. BIO8 was particularly relevant for *A. turkestanicum* (10.4%), whereas BIO15 and BIO4 contributed more substantially to the model of *A. karataviense*. These differences indicate that, although both species share Elevation, Slope, and UVB2 as major environmental predictors, the relative importance of topographic and climatic factors differs between them. Such patterns may also relate to the biological characteristics of *Allium* species, which are geophytes with pronounced seasonal growth cycles (Kamenetsky 1994, 1996). Their development is closely linked to specific periods of temperature and moisture availability, particularly during early spring growth and flowering, which may explain the additional contribution of variables describing temperature and precipitation seasonality.

The response curves provide additional insight into the ecological preferences of both species (Figure 4; Figures S4 and S5). For *A. karataviense*, habitat suitability was highest at relatively low slope values and decreased continuously with increasing slope. Suitability increased with elevation and reached a maximum at approximately 1000 m. *A. karataviense* showed a clear optimum at relatively high UVB2 values, reaching maximum habitat suitability at approximately 2.3 × 10^5^ J/m^2^/day, as well as a preference for moderate temperature seasonality and cooler conditions during the wettest quarter (5°C–10°C). These patterns are consistent with its occurrence in mountainous environments, where topographic and climatic conditions may vary considerably over relatively short distances (Shafer et al. 2005). The positive response to BIO12 suggests that adequate moisture availability may be important for successful growth and reproduction during the relatively short active growing season of this spring geophyte. At the same time, the BIO15 response indicates an association with habitats experiencing moderate seasonal variability in precipitation, whereas both low and high precipitation seasonality were associated with lower habitat suitability. These relationships are consistent with the ecology of spring ephemeroid geophytes, whose growth and flowering depend on sufficient soil moisture during the early growing season (Khodorova and Boitel‐Conti 2013).

In contrast, *A. turkestanicum* exhibited a complex, bimodal response to BIO8, with high habitat suitability at very low values, a sharp decline around −5°C to −0°C, and a secondary increase at approximately 10°C–12°C. This pattern suggests that *A. turkestanicum* can tolerate a relatively broad range of temperature conditions during the wettest quarter, but shows higher suitability under relatively cool conditions, with a secondary optimum at moderate temperatures. The responses to BIO12 and BIO15 further indicated a preference for relatively low to intermediate precipitation conditions, with suitability declining at higher precipitation and precipitation seasonality values. BIO4 showed a generally unimodal response, with highest suitability at approximately 1100–1200, followed by a decline at higher values. These climatic responses, together with its optimal elevation of approximately 500 m, indicate an ecological preference for relatively low‐elevation environments characterized by specific temperature and precipitation regimes.

Predicted current distributions of both species (Figures 5 and 7) align well with their known natural ranges described in floristic and ecological studies (Pavlov 1947; Vesselova et al. 2025; POWO 2026) and further validate the models and support their use for future projections.

Future projections showed contrasting responses of the two species to climate change. *A. karataviense* shows relative stability or even expansion of suitable habitats under most scenarios, particularly under ssp245 and ssp585, where an increase in moderate and highly suitable areas is observed (Table 3, Figure 6). It should also be noted that a substantial proportion of the highly suitable habitats for *A. karataviense* is concentrated in the western mountainous regions of Kyrgyzstan and the Karatau mountains of Kazakhstan. This may be due to moderate warming in the mountains, which can create conditions that are potentially suitable as climatic zones shift. As temperatures increase, areas that were previously too cold or climatically restrictive at higher elevations may become suitable for colonization (Körner and Hiltbrunner 2021). In addition, mountain environments are characterized by high environmental heterogeneity, including strong gradients in elevation, slope, and microclimate (Deák et al. 2021). This heterogeneity provides a wide range of «safe havens» ‐ microrefugia that can buffer species against the negative effects of climate change (Keppel et al. 2012; Bátori et al. 2019). For *A. karataviense*, which is associated with rocky substrates and scree habitats, such microhabitats may remain stable even under changing macroclimatic conditions. This contributes to the limited changes in total suitable area (from 150,515 km^2^ currently to 160,626 km^2^ under ssp585 in the 2090s). However, this pattern should not be interpreted as fully beneficial. The increase in suitable area is partly driven by the expansion of low‐ and moderate‐suitability zones, while highly suitable habitats increase more slowly. This may indicate that new habitats are not fully optimal and support less stable or lower‐density populations. Additionally, upward or spatial shifts in suitable habitats may lead to isolation and fragmentation of populations, particularly in mountainous environments (García‐Fernández et al. 2013).

The highly suitable habitats of *A. turkestanicum* are primarily concentrated in southern Kazakhstan, including the Turkestan and Zhambyl regions, as well as the Karatau and Chu‐Ili mountains, and the Betpak‐Dala and Balkhash‐Alakol areas. *A. turkestanicum* demonstrates a consistent decline in total suitable areas across all scenarios (Table 3, Figure 8). Despite a relative increase in the proportion of highly suitable habitats, the overall contraction suggests increasing habitat fragmentation and potential population decline. The scenario ssp585 can be considered the most unfavorable for *A. turkestanicum* , as it leads to the strongest reduction in total suitable area and the greatest spatial shifts. The ssp126 scenario appears to be the least harmful, with comparatively smaller reductions and more stable distributions. This pattern can be explained by projected large‐scale aridification under the ssp585 scenario. It has been estimated that up to 68.3% of global land areas will experience increased drought, with 93.2% of arid regions undergoing significant drought intensification by the end of the 21st century (Li et al. 2021). Moreover, according to Li et al. (2021), the average global drought duration is expected to increase from 4.4 months (1960–2000) to 8.6 months (2061–2100), indicating a substantial extension of dry periods. Importantly, arid regions, including Central Asia, are projected to become the most drought‐affected under this scenario (Li et al. 2021). Given that *A. turkestanicum* is primarily associated with desert‐steppe and semi‐arid environments, its distribution is already constrained by water availability (Scholes 2020). Further intensification of drought leads to reduced soil moisture, increased evapotranspiration, and degradation of suitable habitats (Li et al. 2021). As a result, large areas currently classified as low or moderately suitable become unsuitable, leading to an overall contraction of the species' range.

The centroid migration analysis further confirmed previous statements. *A. karataviense* exhibited minor centroid movements across all scenarios, indicating a high degree of spatial stability and suggesting that suitable habitats for *A. karataviense* remain within the same geographic region over time. At the same time, *A. turkestanicum* demonstrated greater centroid displacement, reflecting higher sensitivity to climatic changes. The movement toward the northwest suggests migration toward cooler, potentially less arid environmental conditions.

Overall, these results clearly indicate that *A. karataviense* is the more stable species, while *A. turkestanicum* is more vulnerable to environmental change. The limited centroid shifts in *A. karataviense* reflect its association with mountainous environments, where local topographic heterogeneity provides buffering capacity against climate change. In contrast, consistent centroid movements in *A. turkestanicum* highlight vulnerability to increasing aridity, leading to redistribution of suitable habitats over considerable distances.

The overlap between predicted highly suitable habitats and existing SPNTs remains limited for both species, indicating insufficient coverage by the current conservation network. For *A. turkestanicum* , only 12.73% of highly suitable habitats fall within protected areas, whereas for *A. karataviense* this value is even lower at 11.65%. Therefore, there is a clear need to strengthen conservation efforts and expand the existing network of protected areas to ensure more effective coverage of these suitable habitats. The identification of new conservation areas can be guided by the IPA framework. Both studied species meet IPA subcriterion B3: the area contains an exceptional number of socially, economically, or culturally valuable species (Dubynin and Dimeyeva 2025). *A. karataviense* is a significant medicinal plant (Keusgen et al. 2006; Grudzinskaya et al. 2014), while *A. turkestanicum* is a wild relative of the cultivated onion and represents an important genetic resource for food security (Sitpayeva et al. 2020; Crop Wild Relatives Occurrence Data Consortia 2024).

A potential IPA can be proposed where the suitable habitats of both *Allium* species overlap. Based on the modeling results, such a zone is located within the Western Tien Shan, including territories of southern Kazakhstan and neighboring countries. This region represents a key biogeographical intersection, where mountainous habitats suitable for *A. karataviense* change to foothill and steppe environments occupied by *A. turkestanicum* . The Karatau Mountains and Chu‐Ili Mountains can be considered as core areas within this broader zone, as they combine high habitat suitability for both species and maintain secure conditions under future climate scenarios. However, additional studies are needed to confirm the effectiveness and boundaries of this proposed IPA. These areas represent a biodiversity hotspot (Shaimoldina et al. 2026), suggesting that their protection could help conserve a much broader range of valuable species and increase the overall effectiveness of plant conservation efforts.

This study is the first to model the potential distribution of *A. karataviense* and *A. turkestanicum* across Central Asia under both current and future climate scenarios. Although *A. karataviense* and *A. turkestanicum* have been investigated from taxonomic, genetic, biochemical, and floristic perspectives, their potential distributions, climatic niches, and responses to future climate change have not previously been assessed using species distribution modeling (Kadyrbayeva et al. 2021; Dusalieva et al. 2025; Vesselova et al. 2025; Almerekova et al. 2026; Ergashov et al. 2026; Yermagambetova et al. 2026). By comparing two species with contrasting environmental preferences, our study provides new insights into the ecological factors governing their distributions and their potential vulnerability to climate change. Furthermore, identifying areas with high habitat suitability provides a scientific basis for conservation planning, including prioritizing field surveys, assessing the representation of suitable habitats within existing protected areas, and identifying regions that may require additional conservation attention under future climate conditions.

However, the selection of environmental variables was constrained by data availability, particularly the limited availability of high‐resolution soil and other ecologically relevant datasets for Central Asia. Including additional environmental variables, such as sand, clay, and soil moisture, could further improve model performance and provide a more comprehensive understanding of species‐environment relationships. Our models were based primarily on climatic, topographic, soil, and UV‐B variables and therefore do not account for other factors that may influence species distributions. These include biotic interactions (e.g., competition with co‐occurring vegetation, herbivory, pathogens, and pollinator availability), dispersal limitations that may restrict colonization of newly suitable habitats, and anthropogenic pressures such as overgrazing. In addition, future distribution projections are subject to uncertainties associated with global climate models and greenhouse gas emission scenarios. Consequently, the predicted suitable areas should be interpreted as potential rather than guaranteed future distributions (Elith and Leathwick 2009).

## 5 Conclusion

This study provides a comprehensive analysis of the current and future distribution areas of *A. karataviense* and *A. turkestanicum* in Central Asia using MaxEnt modeling. The results revealed clear differences in ecological preferences and climate sensitivity between the two species. *A. karataviense* is primarily associated with mountainous environments, which contribute to its relatively stable distribution under future climate scenarios. In contrast, *A. turkestanicum* is adapted to lowland habitats and is more sensitive to rising temperatures and aridity, resulting in a consistent contraction of its suitable range across all projected scenarios. Future projections indicate that *A. karataviense* may maintain or even expand its suitable habitats, particularly under moderate and high‐emission scenarios, likely due to the availability of microrefugia in heterogeneous mountain landscapes. However, this expansion is mainly driven by moderately suitable areas, suggesting potential limitations in habitat quality. *A. turkestanicum* is expected to experience significant habitat loss and spatial redistribution, with pronounced northwestward shifts, reflecting its vulnerability to ongoing climate change.

The limited overlap between highly suitable habitats and existing protected areas highlights a critical gap in the current conservation network. Only 11.65% and 12.73% of the optimal habitats for *A. karataviense* and *A. turkestanicum* , respectively, are currently protected, underscoring the need to expand and optimize conservation strategies. Based on the modeling results, the Karatau and Chu‐Ili Mountains systems emerge as key priority areas where suitable habitats of both species overlap. These areas represent a biodiversity hotspot and hold strong potential for designation as an IPA, which could enhance the conservation of not only these two species but also a broader range of rare and economically valuable taxa. This study emphasizes the necessity of targeted, climate‐informed conservation actions in Central Asia to ensure the long‐term preservation of ecologically and economically significant *Allium* species.

## Author Contributions

**Alan Ibraimov:** data curation (lead), formal analysis (lead), investigation (equal), software (equal), writing – original draft (lead). **Aruzhan Alikhanova:** data curation (equal), formal analysis (equal), methodology (equal), visualization (equal), writing – review and editing (equal). **Saule Abugalieva:** conceptualization (equal), funding acquisition (equal), project administration (equal), supervision (equal), writing – original draft (equal), writing – review and editing (equal). **Shyryn Almerekova:** conceptualization (equal), supervision (equal), writing – original draft (equal), writing – review and editing (equal).

## Funding

This research was funded by the Science Committee of the Ministry of Science and Higher Education of the Republic of Kazakhstan (AP23490860).

## Conflicts of Interest

The authors declare no conflicts of interest.

## Supporting information


**Figure S1:** Map of occurence points for *Allium karataviense* and *Allium turkestanicum* (Source: GBIF).
**Figure S2:** MaxEnt modeling process.
**Figure S3:** Continuous Boyce curves for: (A) *Allium karataviense* , (B) *Allium turkestanicum* .
**Figure S4:** Response curves of environmental variables for *Allium karataviense* .
**Figure S5:** Response curves of environmental variables for *Allium turkestanicum* .

**Table S1:** Thinned occurrence data of *Allium karataviense* . Source: GBIF.
**Table S2:** Thinned occurrence data of *Allium turkestanicum* . Source: GBIF.
**Table S3:** List of environmental variables used in this study.
**Table S4:** Continuous Boyce Index, habitat suitability thresholds, and segmented regression statistics for *Allium karataviense* and *A. turkestanicum.*

**File S1:** Central Asia shapefile.

**File S2:** Environmental layers.

**File S3:** SPNTs shapefile.

**File S4:** R codes for MaxEnt modeling.

## Data Availability

The data supporting the findings of this study are provided in the Supporting Information (Figures and Tables). The Supporting files used for species distribution modeling have been deposited in the Zenodo repository and are publicly accessible via the following DOI: https://doi.org/10.5281/zenodo.21862306.
