# Supplementary material for: Current and Future Distributions of Allium karataviense and A. turkestanicum in Central Asia Using MaxEnt Modeling
Source: Ecol Evol. 2026 Sep 9;16(9):e74315. doi: 10.1002/ece3.74315 (PMC13557923; doi:10.1002/ece3.74315)
Supplement: Supplementary file 1 [file ECE3-16-e74315-s006.pdf]

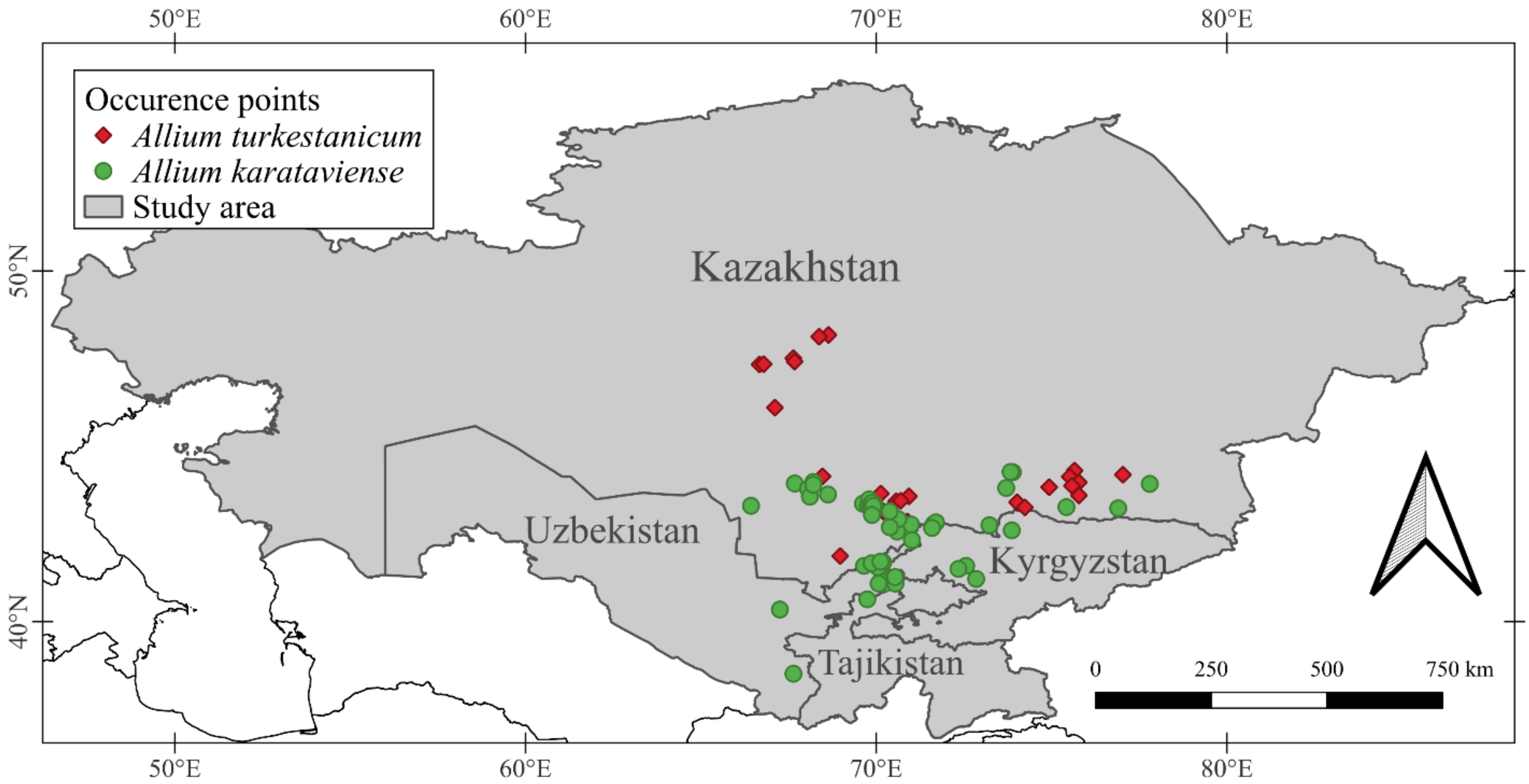

Supplementary Figure S1. Map of occurence points for *Allium karataviense* and *Allium turkestanicum* (Source: GBIF)

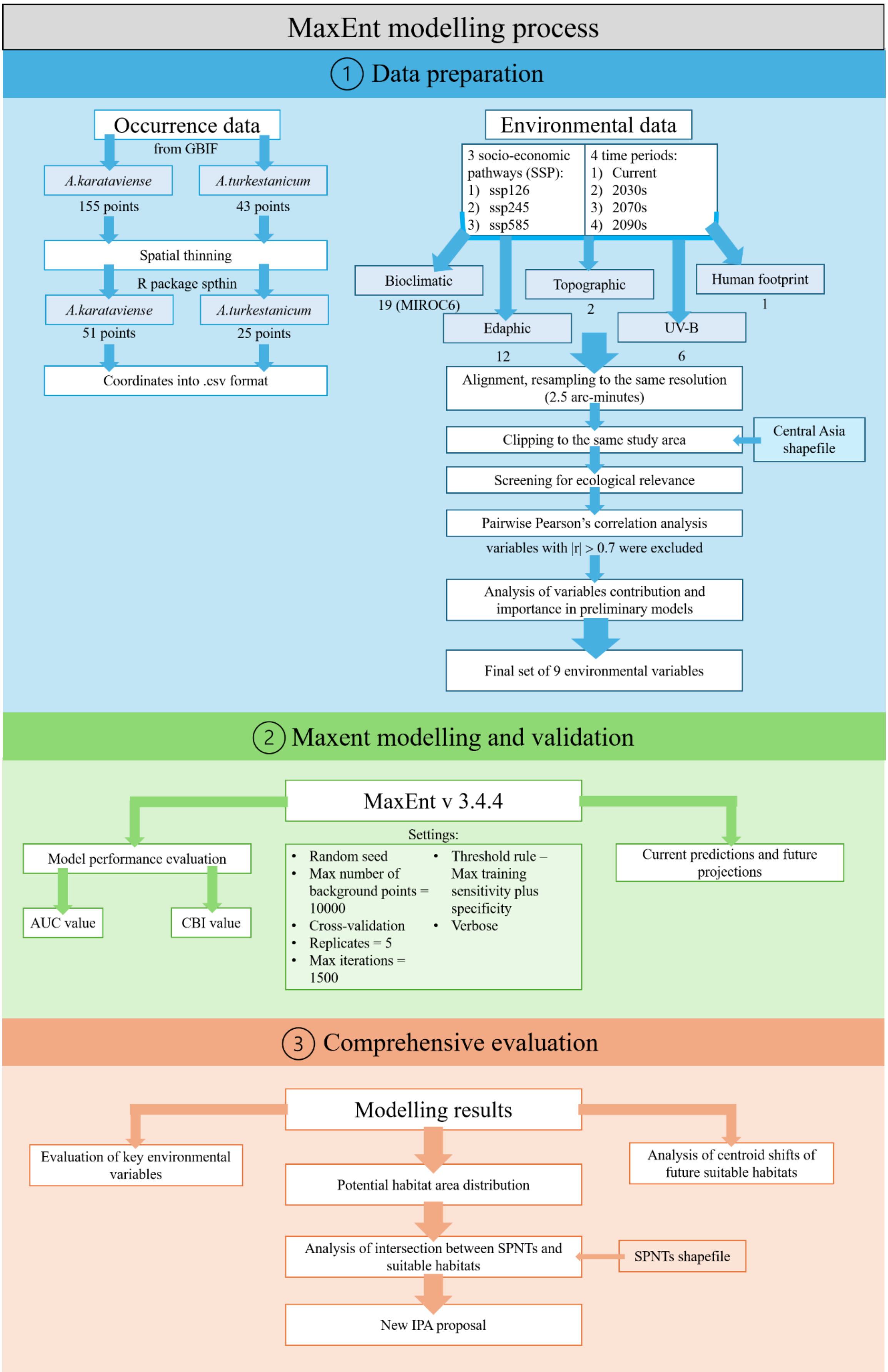

Supplementary Figure S2. MaxEnt modelling process

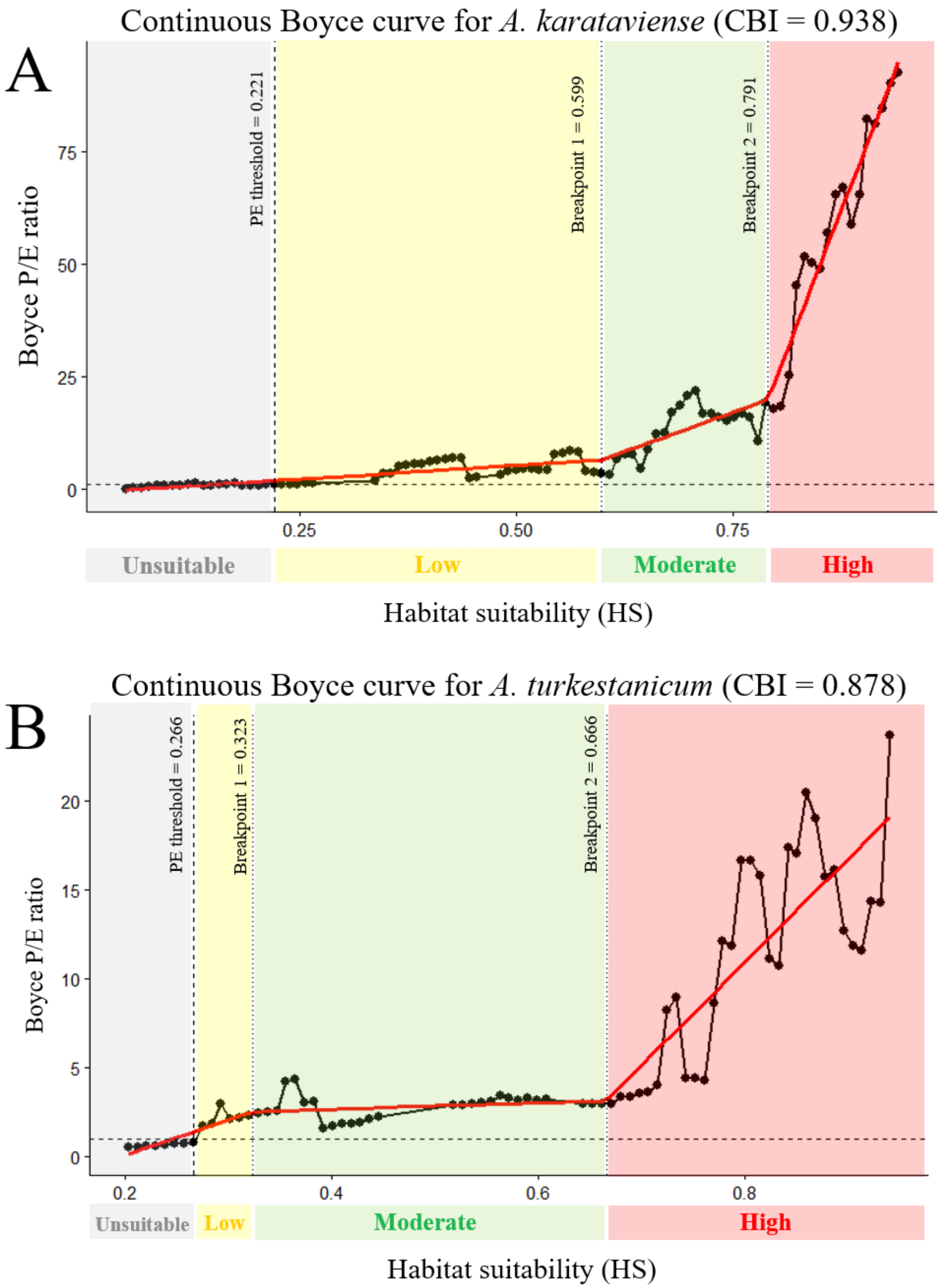

Supplementary Figure S3. Continuous Boyce curves for: (A) *Allium karataviense*, (B) *Allium turkestanicum*

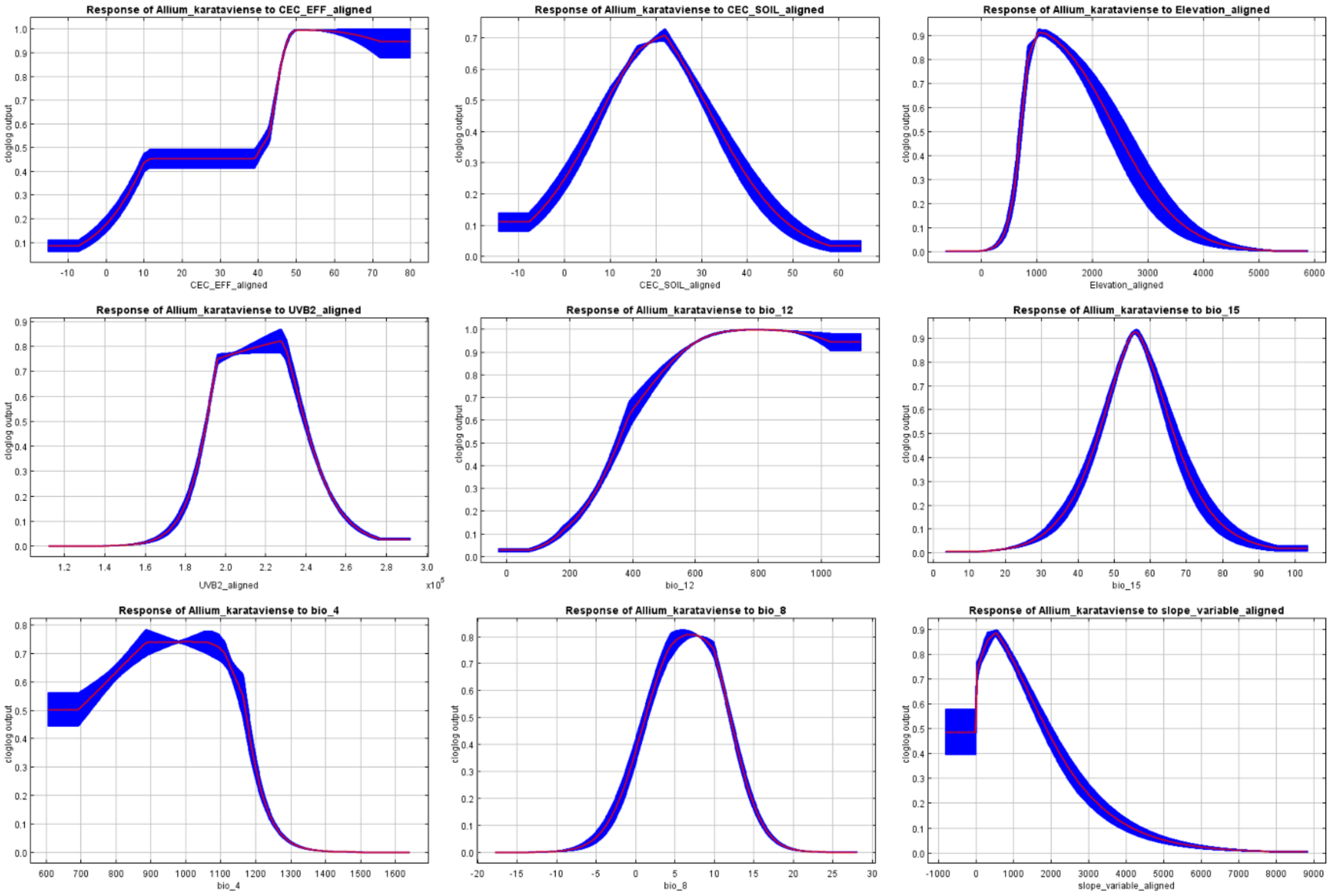

Supplementary Figure S4. Response curves of environmental variables for *Allium karataviense*

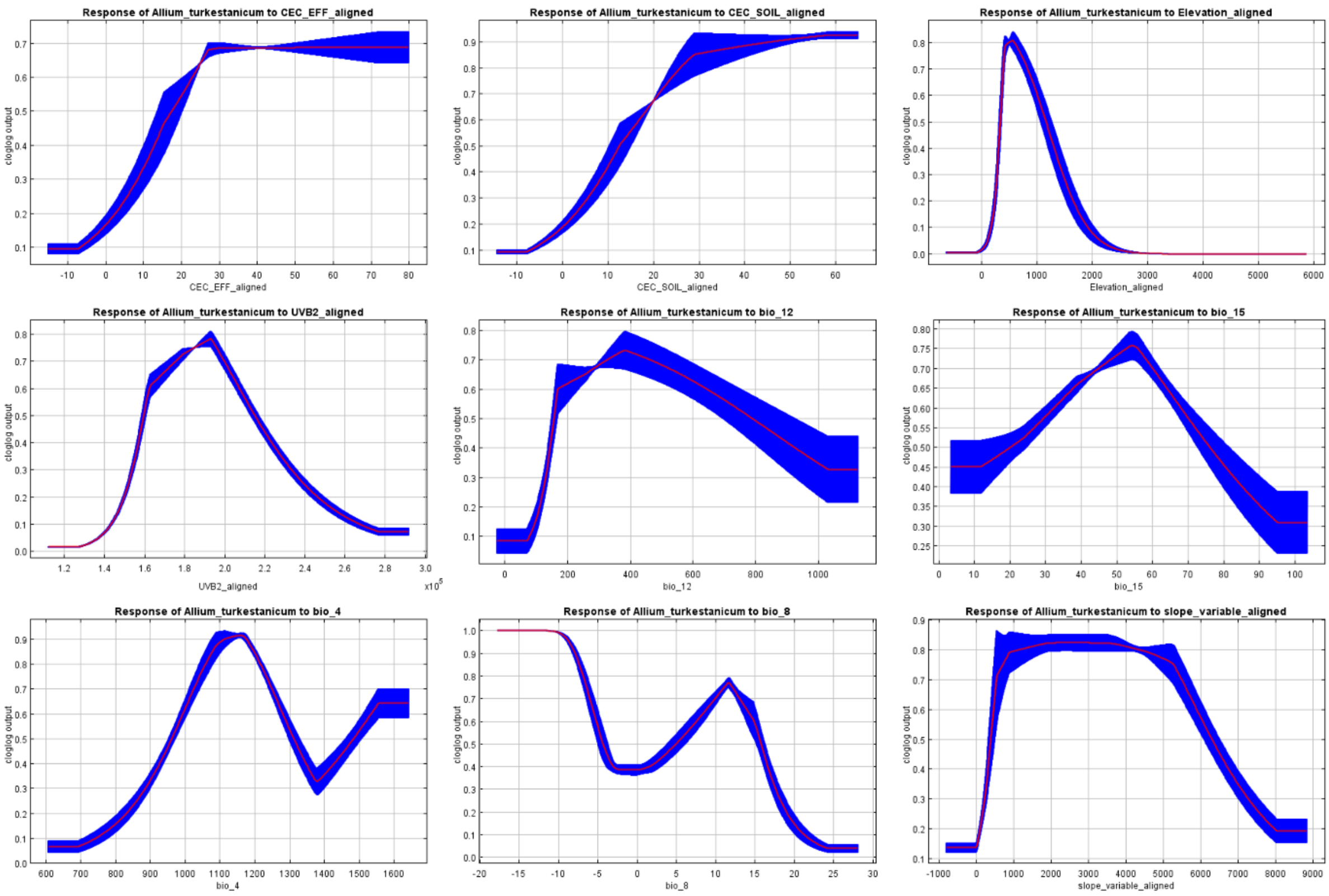

Supplementary Figure S5. Response curves of environmental variables for *Allium turkestanicum*
